# *RESISTANCE TO POWDERY MILDEW8.1* boosts pattern‐triggered immunity against multiple pathogens in Arabidopsis and rice

**DOI:** 10.1111/pbi.12782

**Published:** 2017-07-27

**Authors:** Yan Li, Yong Zhang, Qing‐Xia Wang, Ting‐Ting Wang, Xiao‐Long Cao, Zhi‐Xue Zhao, Sheng‐Li Zhao, Yong‐Ju Xu, Zhi‐Yuan Xiao, Jin‐Lu Li, Jing Fan, Hui Yang, Fu Huang, Shunyuan Xiao, Wen‐Ming Wang

**Affiliations:** ^1^ Rice Research Institute and Key Lab for Major Crop Diseases Sichuan Agricultural University Chengdu China; ^2^ Collaborative Innovation Center for Hybrid Rice in Yangtze River Basin Sichuan Agricultural University Chengdu China; ^3^ College of Agronomy and Key Lab for Major Crop Diseases Sichuan Agricultural University Chengdu China; ^4^ Institute of Bioscience and Biotechnology Research Department of Plant Science and Landscape Architecture University of Maryland College Park MD USA

**Keywords:** *RPW8*, disease resistance, *Pseudomonas syringae*, *Pyricularia oryzae*, *Xanthomonas oryzae*, pattern‐triggered immunity

## Abstract

The Arabidopsis gene *RESISTANCE TO POWDERY MILDEW8.1* (*RPW8.1*) confers resistance to virulent fungal and oomycete pathogens that cause powdery mildew and downy mildew, respectively. However, the underlying mechanism remains unclear. Here, we show that ectopic expression of *RPW8.1* boosts pattern‐triggered immunity (PTI) resulting in enhanced resistance against different pathogens in both Arabidopsis and rice. In Arabidopsis, transcriptome analysis revealed that ectopic expression of *RPW8.1‐YFP* constitutively up‐regulates expression of many pathogen‐associated molecular pattern (PAMP‐)‐inducible genes. Consistently, upon PAMP application, the transgenic line expressing *RPW8.1‐YFP* exhibited more pronounced PTI responses such as callose deposition, production of reactive oxygen species, expression of defence‐related genes and hypersensitive response‐like cell death. Accordingly, the growth of a virulent bacterial pathogen was significantly inhibited in the transgenic lines expressing *RPW8.1‐YFP*. Conversely, impairment of the PTI signalling pathway from PAMP cognition to the immediate downstream relay of phosphorylation abolished or significantly compromised *RPW8.1*‐boosted PTI responses. In rice, heterologous expression of *RPW8.1‐YFP* also led to enhanced resistance to the blast fungus *Pyricularia oryzae* (syn. *Magnaporthe oryzae*) and the bacterial pathogen *Xanthomonas oryzae* pv. *oryzae* (Xoo). Taken together, our data suggest a surprising mechanistic connection between *RPW8.1* function and PTI, and demonstrate the potential of *RPW8.1* as a transgene for engineering disease resistance across wide taxonomic lineages of plants.

## Introduction

Plants are equipped with various cell surface‐localized pattern‐recognition receptors (PRRs) to detect pathogen‐associated molecular patterns (PAMPs) and activate defence responses termed PAMP‐triggered immunity (PTI) (Jones and Dangl, 2006). Typical defence responses in PTI include the activation of mitogen‐activated protein kinases (MAPKs), burst of reactive oxygen species (ROS), callose deposition and expression of immune‐related genes (Boller and Felix, 2009). Flg22 and chitin are two PAMPs frequently used in PTI‐related studies. Flg22 is a conserved 22‐amino acid peptide derived from bacterial flagella, and perceived by Flagellin Sensing2 (FLS2) in Arabidopsis and OsFLS2 in rice (Felix *et al*., 1999; Gomez‐Gomez and Boller, 2000; Takai *et al*., 2008). Chitin is a fungal cell wall‐derived PAMP perceived by the receptor‐like kinase chitin elicitor receptor kinase1 (CERK1) in Arabidopsis and OsCERK1 together with chitin oligosaccharide elicitor‐binding protein (OsCEBiP) in rice (Kaku *et al*., 2006; Miya *et al*., 2007). To date, several key components in PTI signalling have been identified. These include BRI1‐ASSOCIATED RECEPTOR KINASE1 (BAK1), a coreceptor that interacts with FLS2 to recognize flagella to initiate PTI (Chinchilla *et al*., 2007; Heese *et al*., 2007), and BOTRYTIS‐INDUCED KINASE1 (BIK1), a member of the AvrPphB susceptible1 (PBS1)‐like (PBL) protein family that relays the signal from FLS2 and BAK1 through phosphorylation (Lu *et al*., 2010; Zhang *et al*., 2010). Perception of flg22 by FLS2/BAK1 in Arabidopsis leads to activation of BIK1 and several other PBL family members including PBL1, PBL2 and PBS1, which in turn triggers ROS burst and other defence responses (Kadota *et al*., 2014; Li *et al*., 2014a).

Adapted pathogens subvert PTI by effector repertoires that target components of PTI signalling, thereby establishing the so‐called effector‐triggered susceptibility (ETS) (Jones and Dangl, 2006). For example, the well‐studied *Pseudomonas syringae* effector AvrPto targets the PRRs FLS2 and EFR to block PTI in Arabidopsis (Xiang *et al*., 2008). Another effector, HopAi1, targets the PTI signalling components MPK3 and MPK6 to compromise defence responses (Zhang *et al*., 2007). In turn, plants exploit resistance (R) proteins to recognize effectors to activate a stronger defence programme, called effector‐triggered immunity (ETI), to mount effective resistance. ETI is usually culminated in the hypersensitive response (HR), a rapid programmed cell death confined to the site of infection (Jones and Dangl, 2006). In some cases, however, defence responses in PTI and ETI may be indistinguishable (Thomma *et al*., 2011). Most of the identified R proteins are structurally conserved with a nucleotide‐binding site (NBS) and leucine‐rich repeats (LRR), and act as intracellular immune receptors to directly or indirectly recognize their cognate effectors (Bonardi *et al*., 2012). Some other genetically defined R proteins are cell surface‐localized receptor‐like transmembrane proteins (RLPs) or receptor‐like kinases (RLKs) (Dangl and Jones, 2001).

Some *PRR* and *R* genes have been demonstrated to function as transgenes across wide taxonomic lineages of plants, including from a dicot to a monocot, from a monocot to a dicot and between two different dicots. The Arabidopsis *PRR* gene *EFR* conferred enhanced resistance to different bacterial pathogens when expressed in *Nicotiana benthamiana* or tomato (Lacombe *et al*., 2010). Transgenic rice expressing *EFR* also showed broad‐spectrum resistance to bacterial pathogens (Schwessinger *et al*., 2015). Ectopic expression of the wheat resistance gene *Lr34* conferred blast resistance in rice and leaf blight resistance in maize (Krattinger *et al*., 2016; Sucher *et al*., 2017). The barley powdery mildew resistance genes *MLA1* and *MLA13* retained their ability to recognize their cognate effectors from barley powdery mildew in transgenic Arabidopsis plants (Maekawa *et al*., 2012). However, it remains an open question whether *R* genes from a dicot can activate resistance in a monocot.

*RPW8.1* and *RPW8.2* (hereafter referred to as *RPW8* unless otherwise indicated) are two homologous genes that confer broad‐spectrum resistance to powdery mildew pathogens in Arabidopsis ecotype Ms‐0 (Xiao *et al*., 2001). While these two genes are tandemly located in the *RPW8* locus in Ms‐0, they are absent from the powdery mildew‐susceptible ecotype Col‐0 (Xiao *et al*., 2004). Thus, Col‐0 is ideal for functional analysis of *RPW8.1* and *RPW8.2* through transgenics. In previous studies, we constructed Arabidopsis transgenic lines expressing RPW8.1‐yellow fluorescent protein (YFP) and RPW8.2‐YFP from their native promoters in Col‐*gl* (Col‐0 containing the glabrous mutation) (Wang *et al*., 2007, 2009). Using these transgenic lines, we found that RPW8.2‐YFP is induced in leaf epidermal cells invaded by the haustorium, the feeding organ of powdery mildew, and specifically targeted to the extrahaustorial membrane (EHM) where it activates resistance to powdery mildew (Wang *et al*., 2009). By contrast, ectopic expression of RPW8.1‐YFP from the *RPW8.1* promoter in Col‐*gl* results in enhanced resistance to both powdery mildew and oomycete pathogens (Ma *et al*., 2014). This interesting observation has thus prompted us to investigate the mechanism underlying *RPW8.1‐YFP*‐mediated resistance.

In this study, we examined the responses of the transgenic Arabidopsis lines expressing *RPW8.1‐YFP* or *RPW8.2‐YFP* to different PAMPs and bacterial strains, and the infection phenotypes of the *RPW8.1‐YFP* transgenic rice lines to fungal and bacterial pathogens. Collectively, our data demonstrate that ectopic expression of *RPW8.1* leads to enhanced PTI signalling, explaining the broad‐spectrum resistance mediated by *RPW8.1*, and suggest that *RPW8.1* could be exploited for engineering resistance in crops such as rice.

## Results

### *RPW8.1*, but not *RPW8.2*, enhances resistance to bacterial pathogens in Arabidopsis

**Figure 1:**
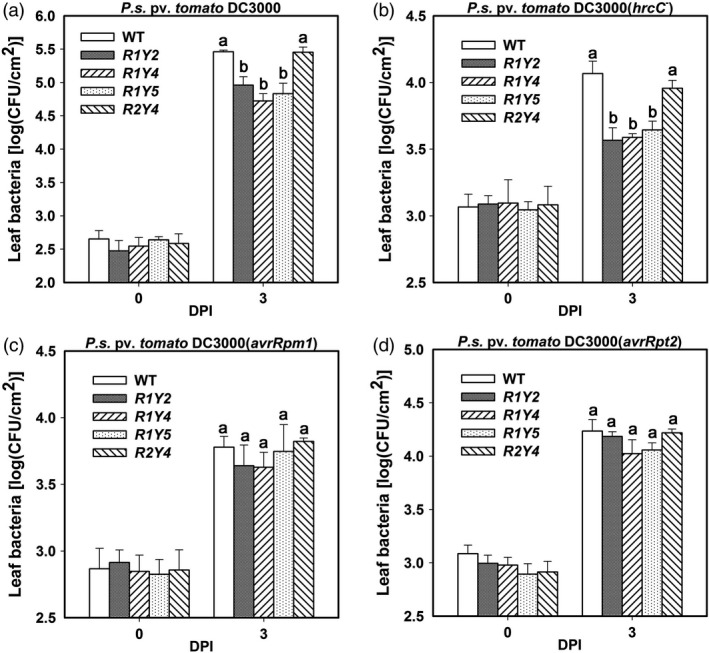
Ectopic expression of *RPW8.1‐YFP* enhances resistance to bacterial pathogens in Arabidopsis. (a‐d) Bacterial growth assay for the indicated strains in the indicated transgenic lines in comparison with the wild‐type (WT) Col‐*gl*. Error bars indicate standard deviation (SD,* n *= 6). Different letters above the bars indicate significant differences (*P *< 0.01) as determined by a one‐way ANOVA followed by post hoc Tukey HSD analysis. Similar results were obtained in three independent experiments.

Previously, we found that ectopic expression of *RPW8.1* leads to enhanced resistance to both powdery mildew and downy mildew, while ectopic expression of *RPW8.2* enhances resistance to only powdery mildew (Ma *et al*., 2014; Wang *et al*., 2007, 2009). These observations prompted us to examine the response of *RPW8.1* and *RPW8.2* transgenic lines to different strains of *P. syringae*. Our data showed that the multiplications of both the virulent strain *P. syringae* DC3000 and the nonpathogenic mutant strain *P. syringae* DC3000(*hrcC* ^*‐*^) in all three *RPW8.1* transgenic lines (i.e. R1Y2, R1Y4 and R1Y5) were significantly lower than that in wild‐type (WT) Col‐*gl* plants, while there was no significant difference between the RPW8.2 transgenic line R2Y4 and WT plants (Figure 1a,b). However, there were only marginal differences between *RPW8.1*,* RPW8.2* transgenic lines and WT in multiplication of both the avirulent strains *P. syringae* DC3000(*avrRpm1*) and *P. syringae* DC3000(*avrRpt2*) (Figure 1c,d). These observations suggest that *RPW8.1*, but not *RPW8.2*, might enhance PTI to improve resistance against the virulent bacterial strain and further restrict the growth of the nonpathogenic strain, whereas neither *RPW8.1* nor *RPW8.2* has impact on ETI in Arabidopsis.

### Ectopic expression of *RPW8.1* constitutively up‐regulates the expression of many PAMP‐inducible genes

To investigate whether *RPW8.1*'s action is connected with PTI signalling, we first examined the expression of *RPW8* after treatment of flg22 or chitin in the Arabidopsis accessions Shahdara and Wa that contain the wild‐type *RPW8* alleles, and the accession Ws that contains nonfunctional alleles of both *RPW8* and the flg22 receptor *FLS2* (Gomez‐Gomez *et al*., 1999; Orgil *et al*., 2007). Results from quantitative RT‐PCR (qRT‐PCR) showed that *RPW8.1* was induced in all the three accessions upon application of flg22 or chitin with the exception that *RPW8.1* was not induced by flg22 in Ws (Figure S1a). Interestingly, the expression of *RPW8.2* also showed PAMP‐induced patterns similar to that of *RPW8.1* (Figure S1b). Consistently, both RPW8.1‐YFP and RPW8.2‐YFP were induced in the respective transgenic lines upon flg22 or chitin treatment (Figure S1c–f). These results suggest that both *RPW8.1* and *RPW8.2* are positively regulated by PTI at the transcription level.

**Figure 2:**
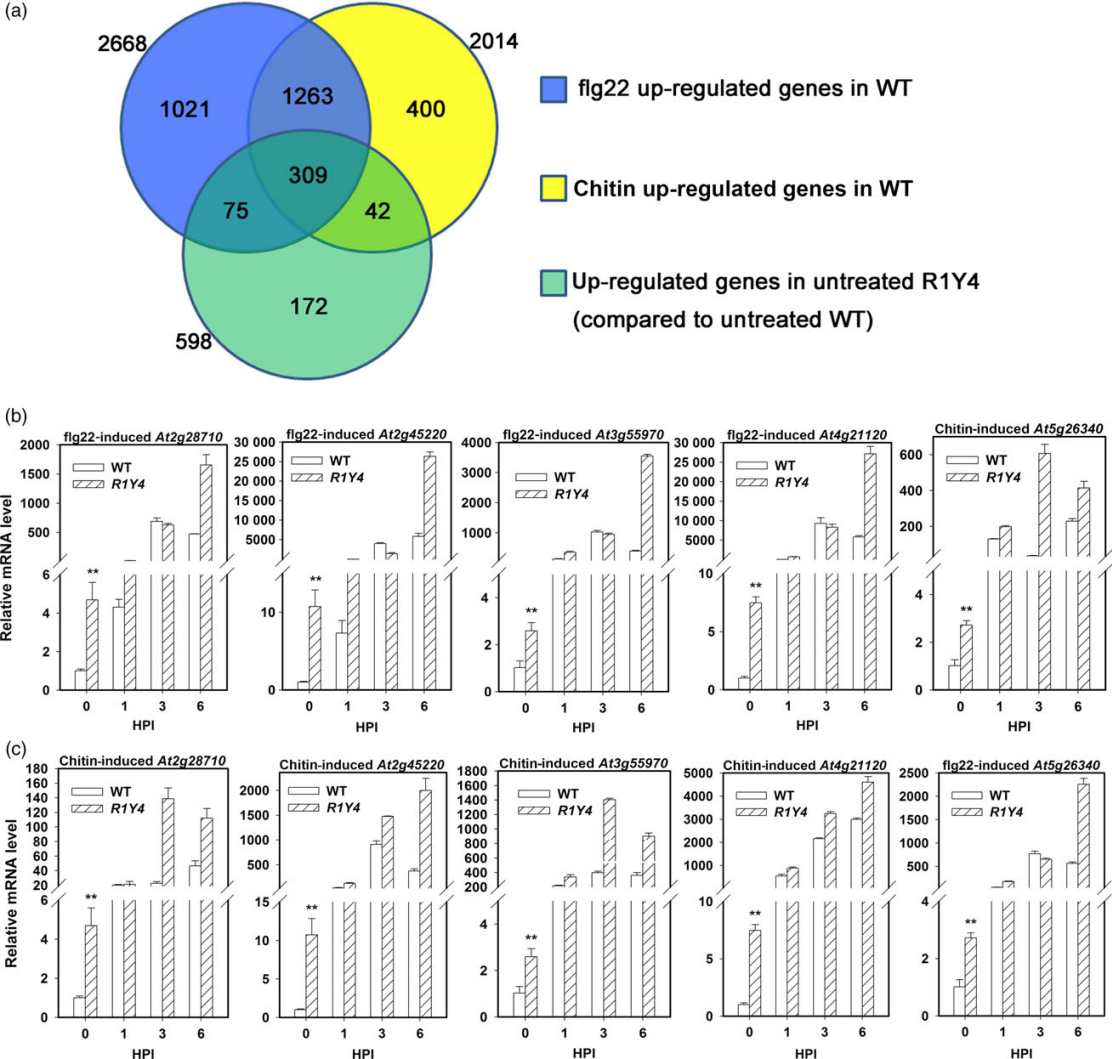
Ectopic expression of *RPW8.1‐YFP* constitutively up‐regulates the expression of PAMP‐inducible genes in Arabidopsis. (a) Comparison of gene numbers between PAMP‐inducible genes in wild‐type (WT) Col‐*gl* and constitutively up‐regulated genes in R1Y4. (b, c) Quantitative RT‐PCR data show that relative mRNA levels of the indicated genes upon application of flg22 (b) and chitin (c) at the indicated time points. Error bars indicate SD (*n *= 3). Student's *t*‐test was carried out to determine the significance of difference between WT and R1Y4 at 0 h postinfiltration (HPI). Asterisks (**) indicated significant difference at *P *≤ 0.01. Similar results were obtained in two independent experiments.

Next, we examined whether there are a common set of genes regulated by PAMPs and ectopic expression of *RPW8.1* through RNA‐seq analysis. Compared to untreated WT plants, more than 2000 genes were up‐regulated in WT plants treated by flg22 or chitin, whereas 598 genes displayed constitutive up‐regulation in untreated R1Y4 in comparison with untreated WT (Figure 2a). Among the up‐regulated genes in R1Y4, 384 (64.2%) genes were also up‐regulated by flg22, 351 (58.7%) genes were up‐regulated by chitin, and 309 (51.7%) genes were consensually induced by flg22 and chitin in WT (Figure 2a, Table S1). Gene Ontology (GO) assay revealed that these 309 genes were responsive to pathogens or involved in defence‐related hormone signalling (Table S2). To validate the RNA‐seq data, five genes were selected (highlighted green in Table S1) for expression analysis using qRT‐PCR. Consistent with the RNA‐seq data, all of the five genes were constitutively up‐regulated in R1Y4 (Figure 2b, c at 0 HPI). Moreover, these five genes were also up‐regulated in WT and further up‐regulated in R1Y4 by flg22 or chitin (Figure 2b,c). These data demonstrate that expression of *RPW8.1* leads to enhanced transcription of many PAMP‐inducible genes.

To further determine whether expression of *RPW8.1* can enhance PTI signalling, we examined the expression of *PRR* genes and other components in PTI signalling by qRT‐PCR upon PAMP application. Intriguingly, the relative mRNA levels of all of the tested *PRR*s and the *PBL*s, including *FLS2*,* CERK1*,* BAK1*,* BIK1*,* PBL1* and *PBL2,* were induced to higher levels in R1Y4 than in WT by flg22 or chitin (Figure S2a,b). Thus, expression of *RPW8.1* can indeed amplify PTI signalling by enhancing the expression of *PRR*s and *PBL*s upon PAMP perception by certain PRRs.

### Ectopic expression of *RPW8.1* heightens PAMP‐triggered defence responses in Arabidopsis

**Figure 3:**
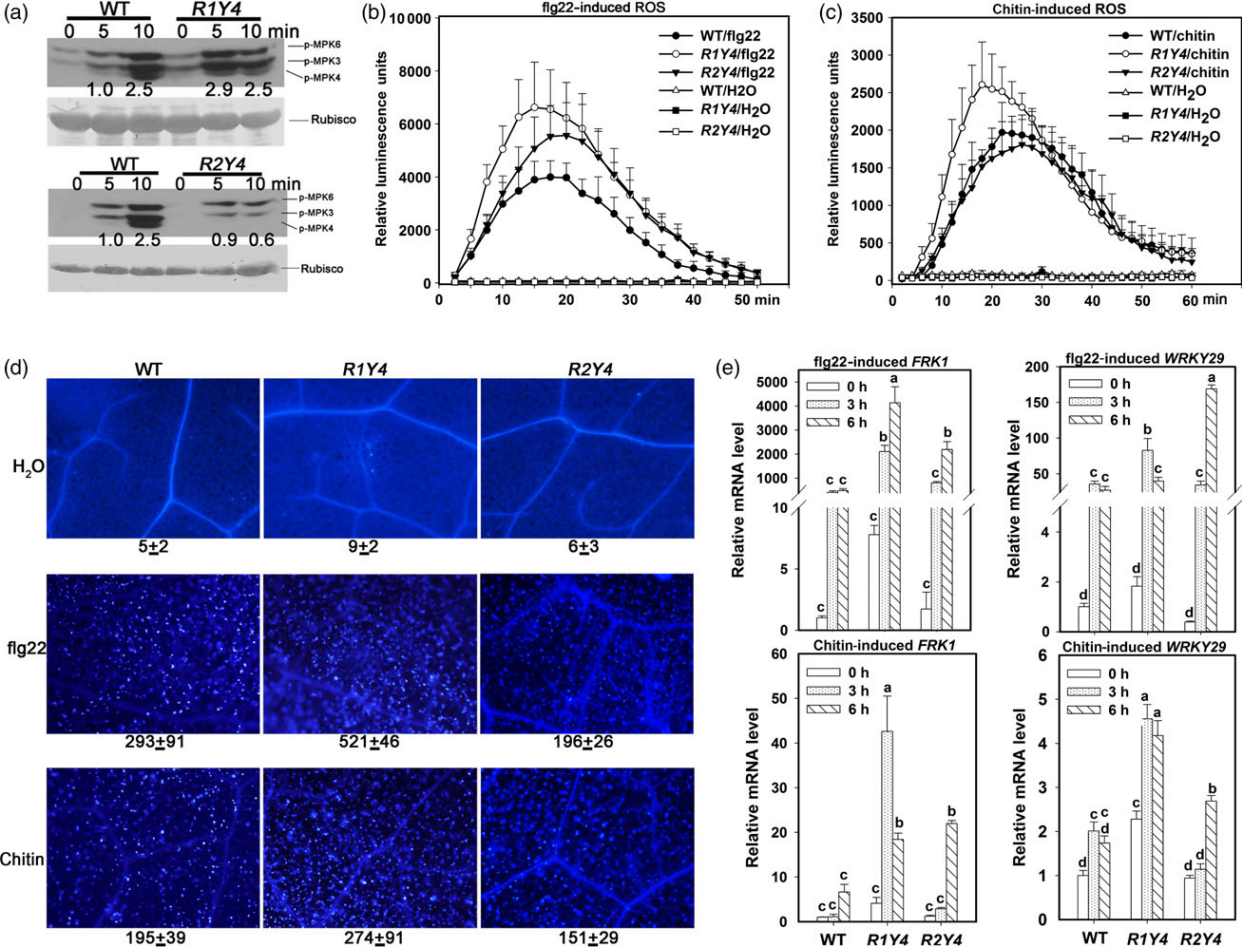
Ectopic expression of *RPW8.1‐YFP* results in enhanced PAMP‐induced defence responses. (a) Western blot analysis shows MAPK activation in wild‐type (WT) Col‐*gl*, R1Y4 and R2Y4 by flg22 at the indicated time points. Phosphorylated MAPKs were detected by anti‐pERK sera. Ponceau S‐stained rubisco was used as loading control. (b, c) Comparison of PAMP‐induced burst of reactive oxygen species (ROS) in WT, R1Y4 and R2Y4. Error bars indicate SD (*n *= 4). (d) Comparison of PAMP‐induced callose deposition in WT, R1Y4 and R2Y4. (e) Quantitative RT‐PCR data show the expression pattern of the indicated PTI marker genes in WT, R1Y4 and R2Y4 upon application of flg22 or chitin. Relative mRNA level was normalized to that in WT at 0 hr. Error bars indicate SD (*n *= 3). Different letters above the bars indicate significant differences at *P *< 0.01. All the experiments were repeated two times with similar results.

To further test whether ectopic expression of *RPW8* boosts PTI, we examined some typical PTI responses. Compared to WT, R1Y4 displayed higher phosphorylated levels of MPK3 and MPK6 at 5 minutes after flg22 treatment, while R2Y4 displayed similar levels at 5 minutes but obviously lower levels at 10 minutes (Figure 3a). Similarly, R1Y4 showed faster and higher levels of ROS production compared to WT upon flg22 or chitin application (Figure 3b,c). R2Y4 also displayed slightly higher levels of ROS accumulation after flg22 treatment but similar levels after chitin application (Figure 3b,c). In addition, R1Y4 displayed significantly more callose deposition than WT, while R2Y4 showed levels slightly lower than WT after treatment with flg22 and chitin (Figure 3d). Consistently, the expression of *FRK1* and *WRKY29* were induced earlier and elevated to significantly higher levels in R1Y4 than that in WT (Figure 3e). Intriguingly, R2Y4 also displayed higher transcription levels of these two genes (Figure 3e), although R2Y4 did not exhibit enhanced resistance to the bacterial strains (Figure 1).

**Figure 4:**
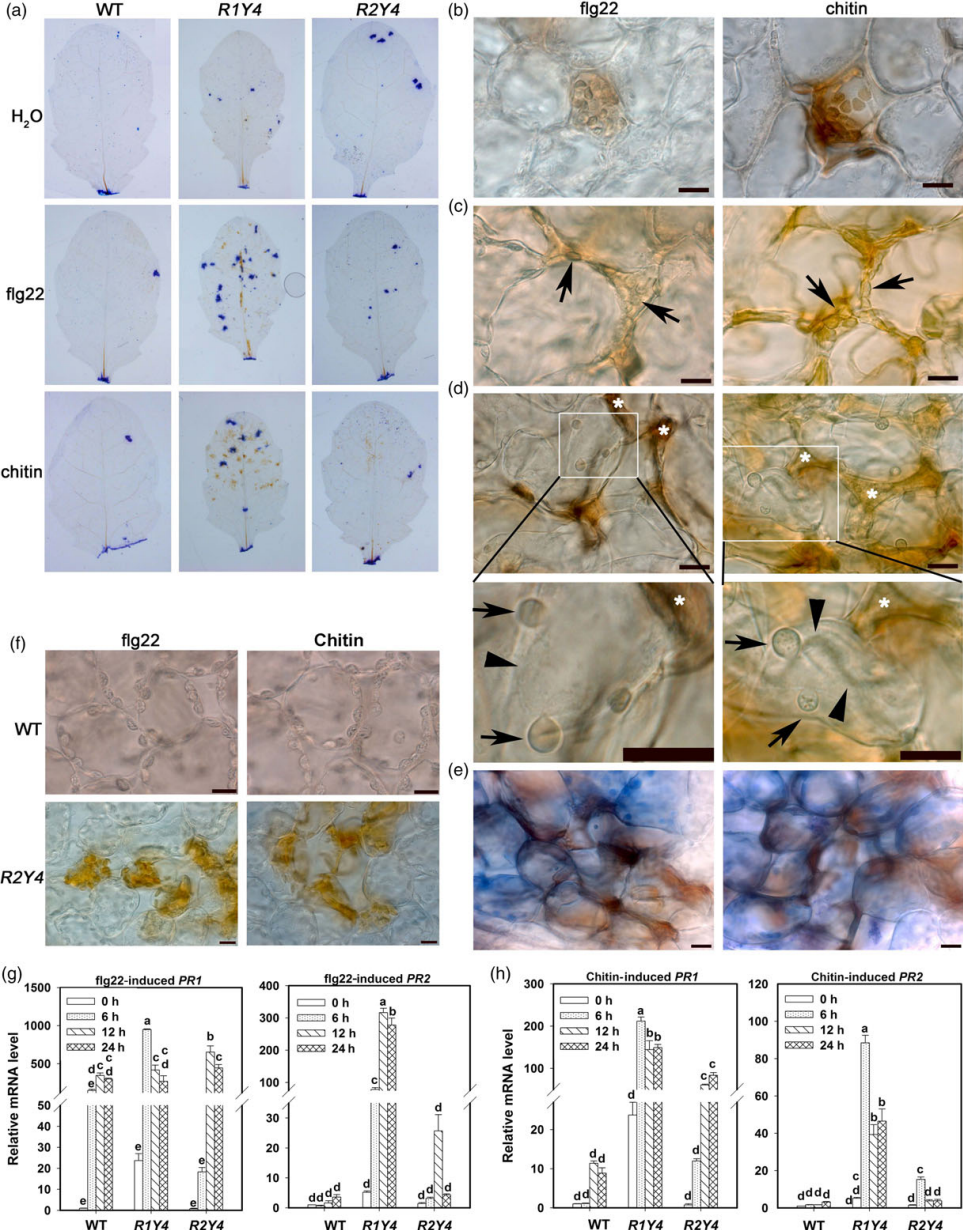
Ectopic expression of *RPW8.1‐YFP* results in PAMP‐induced ETI‐like responses. (a) DAB ‐(3,3’‐diaminobenzidine‐) and trypan blue‐stained leaves from the indicated lines show H_2_O_2_ production and dead cells, respectively. (b‐e) Micrographs show flg22/chitin‐induced ETI‐like responses in R1Y4. Note that during early period of PAMP treatment, H_2_O_2_ was enriched in cells, especially in chloroplasts, and the cells were intact (b). Then, the H_2_O_2_‐accumulated cells were shrunken in the apoplastic space along the neighbouring cells, but the chloroplasts (arrows) were still visible (c). Finally, bubbles (arrows) from the shrinking cytoplasm (arrowheads) were formed in some neighbouring cells of the shrunken cells (*) (d), and eventually formed clusters of dead cells as demonstrated by trypan blue staining (e). Size bar, 10 μm. (f) Micrographs show flg22‐/chitin‐induced H_2_O_2_ in WT and R2Y4 cells. Note that flg22‐ and chitin‐induced H_2_O_2_ accumulation and cell death were not observed in WT cells, but occasionally observed in R2Y4 cells. (g, h) Expression pattern of defence‐related genes *PR1* and *PR2* in the indicated lines upon application of flg22 (g) and chitin (h). Relative mRNA levels were normalized to that in WT at 0 hr. Error bars indicate SD (*n *= 3). Different letters above the bars indicate significant differences at *P *< 0.01. Similar results were obtained in two independent experiments.

In addition, we detected H_2_O_2_ production, HR‐like cell death and the expression of *Pathogenesis‐related* (*PR*) genes in R1Y4 plants induced by flg22 or chitin. Upon PAMP application, H_2_O_2_ accumulation and clusters of dead cells were often observed in R1Y4, but they were rarely seen in R2Y4 and WT (Figure 4a). Detailed microscopic examinations demonstrated that HR‐like cell death was elicited upon PAMP application in R1Y4 (Figure 4b–e). PAMP‐induced H_2_O_2_ accumulation was first detectable in the chloroplasts of one or several individual cells (Figure 4b). Then, such H_2_O_2_‐accumulating cells were shrunken in the apoplast among the neighbouring cells, but the chloroplasts were still visible (Figure 4c). Later on, the chloroplasts disappeared and the cells disintegrated in the apoplastic space among neighbouring cells, which might further trigger death of neighbouring cells as indicated by the bubbling of their cytoplasm (Figure 4d). Eventually, clusters of dead cells were observed (Figure 4e). On the contrary, PAMP‐induced cell death and H_2_O_2_ accumulation were not observed in WT, but occasionally seen in mesophyll cells of R2Y4 (Figure 4f); however, cell shrinkage or bubbling of the cytoplasm was not observed in either WT or R2Y4 (Figure 4a,f). The relative mRNA levels of *PR1* and *PR2* in R1Y4 were induced to significantly higher levels than those in WT (Figure 4g,h). Intriguingly, similar to the expression of *FRK1* and *WRKY29*, the expression of *PR1* and *PR2* was also significantly induced by flg22 or chitin to higher levels in R2Y4 than in WT at 6 h and/or 12 h postinfiltration (HPI), albeit they were not as high as those in R1Y4 (Figure 4g,h).

These results demonstrate that *RPW8.1*, but not *RPW8.2*, can heighten PAMP‐induced defence responses.

### Ectopic expression of *RPW8.1* activates multiple layers of defence responses against virulent bacterial pathogens

**Figure 5:**
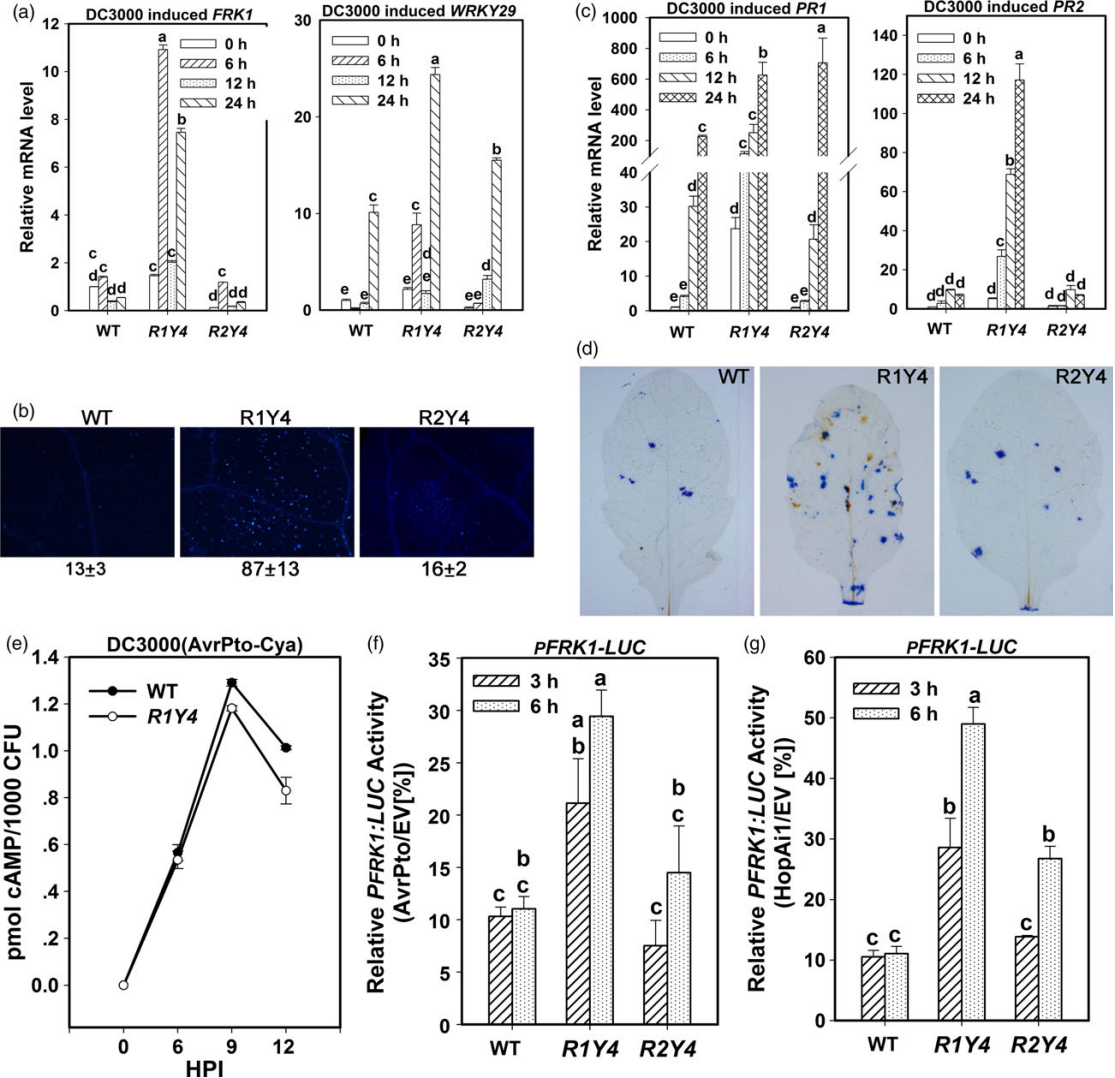
Ectopic expression of *RPW8.1‐YFP* activates multiple layers of defence responses to a virulent bacterial pathogen. (a, c) Quantitative RT‐PCR analysis on the expression of the indicated marker genes upon infection of *P. syringae* DC3000. Error bars indicate SD (*n *= 3). Different letters above the bars indicate significant differences at *P *< 0.01. Similar results were obtained in two independent experiments. (b, d) *P. syringae* DC3000 induced callose deposition (b), H_2_O_2_ production and cell death (d) in the indicated lines revealed by aniline blue, DAB and trypan blue staining, respectively. (e) Adenylate cyclase activity assay shows the different capability of effector secretion in WT and R1Y4 upon *P. syringae* DC3000 infection. The cAMP accumulation was normalized to the bacterial colony numbers at the indicated hours after infiltration (HPI). (f, g) Comparison of AvrPto‐ or HopAi1‐mediated suppression on flg22‐induced *FRK1::LUC* expression. The LUC reporter activity (%) was normalized to the activity of empty vector. Values were normalized to the internal control *35S::RLUC*. Error bars indicate SD (*n *= 3). Different letters above the bars indicate significant differences at *P *< 0.01. Similar results were obtained in three independent experiments.

To address why the expression of *RPW8.1*, but not *RPW8.2* can enhance resistance to the virulent bacterial strain *P. syringae* DC3000, we compared defence responses of R1Y4 and R2Y4 upon infection of this strain. Although expression of both RPW8.1‐YFP and RPW8.2‐YFP was induced in R1Y4 and R2Y4 by *P. syringae* DC3000 (Figure S3), all the canonical PTI responses were only observed in R1Y4. While both *FRK1* and *WRKY29* were significantly induced in R1Y4, only *WRKY29* was induced in R2Y4 (Figure 5a), which agrees with its induction in R2Y4 by PAMP application (Figure 3e), and *FRK1* was not induced in WT and R2Y4 (Figure 5a). Callose deposition was obviously induced in R1Y4, but rarely found in WT and R2Y4 (Figure 5b). Both *PR1* and *PR2* were constitutively expressed and were further up‐regulated to higher levels in R1Y4 than those in WT plants (Figure 5c). By contrast, although *PR1* was induced in R2Y4 to a very high level at 24 HPI, the expression level of *PR2* was comparable to those in WT at all tested time points (Figure 5c). Furthermore, H_2_O_2_ accumulation and clusters of dead cells were observed more frequently in R1Y4 than in WT, whereas there was no apparent difference between R2Y4 and WT plants (Figure 5d).

Given that expression of *RPW8.1* confers enhanced resistance to the virulent bacterial strain *P. syringae* DC3000, we hypothesized that the expression of *RPW8.1* may counteract bacterial virulence on suppressing PTI. Because *P. syringae* DC3000 secretes a suite of effectors to block PTI responses (Cunnac *et al*., 2009), we first exploited a calmodulin‐dependent adenylate cyclase (Cya) reporter system (Schechter *et al*., 2004) to examine whether expression of *RPW8.1* could interfere with effector secretion. In this experiment, to avoid the difference caused by less entry of pathogen into the host cells of R1Y4, we used half of the treated leaves to measure the propagation of bacteria and the other half to measure the activity of adenylate cyclase. Our data showed that the normalized cAMP accumulation per 1000 colonies of bacteria in R1Y4 displayed 10‐20% decrease at 9 and 12 HPI compared to that in WT (Figure 5e), indicating that the inhibition of effector secretion is due to expression of *RPW8.1*. Second, we analysed transcriptional suppression of *FRK1* reporter by AvrPto and HopAi1, two well‐studied *P. syringae* type III virulent effectors (Li *et al*., 2005), using a dual‐luciferase reporter assay (Zhang *et al*., 2010). Flg22‐induced expression of *FRK1* reporter in AvrPto‐ or HopAi1‐expressing WT protoplasts was suppressed to 10% of that in control WT protoplasts at 3 and 6 HPI, whereas such suppression in R1Y4 protoplasts was significantly lessened with the expression level being 20–25% of that in control R1Y4 protoplasts at 3 HPI and 30‐50% at 6 HPI (Figure 5f,g). Interestingly, although the *FRK1* reporter at 3 HPI in R2Y4 displayed a similar level of suppression as that in WT, the expression level at 6 HPI was significantly higher than that in HopAi1‐expressing WT protoplasts (Figure 5g), implying that the expression of *RPW8.2* might also repress virulence of certain effectors.

These results indicate that ectopic expression of *RPW8.1* might activate multiple layers of defence to counteract the infection of virulent bacteria.

### PTI signalling is required for *RPW8.1*‐mediated immunity

**Figure 6:**
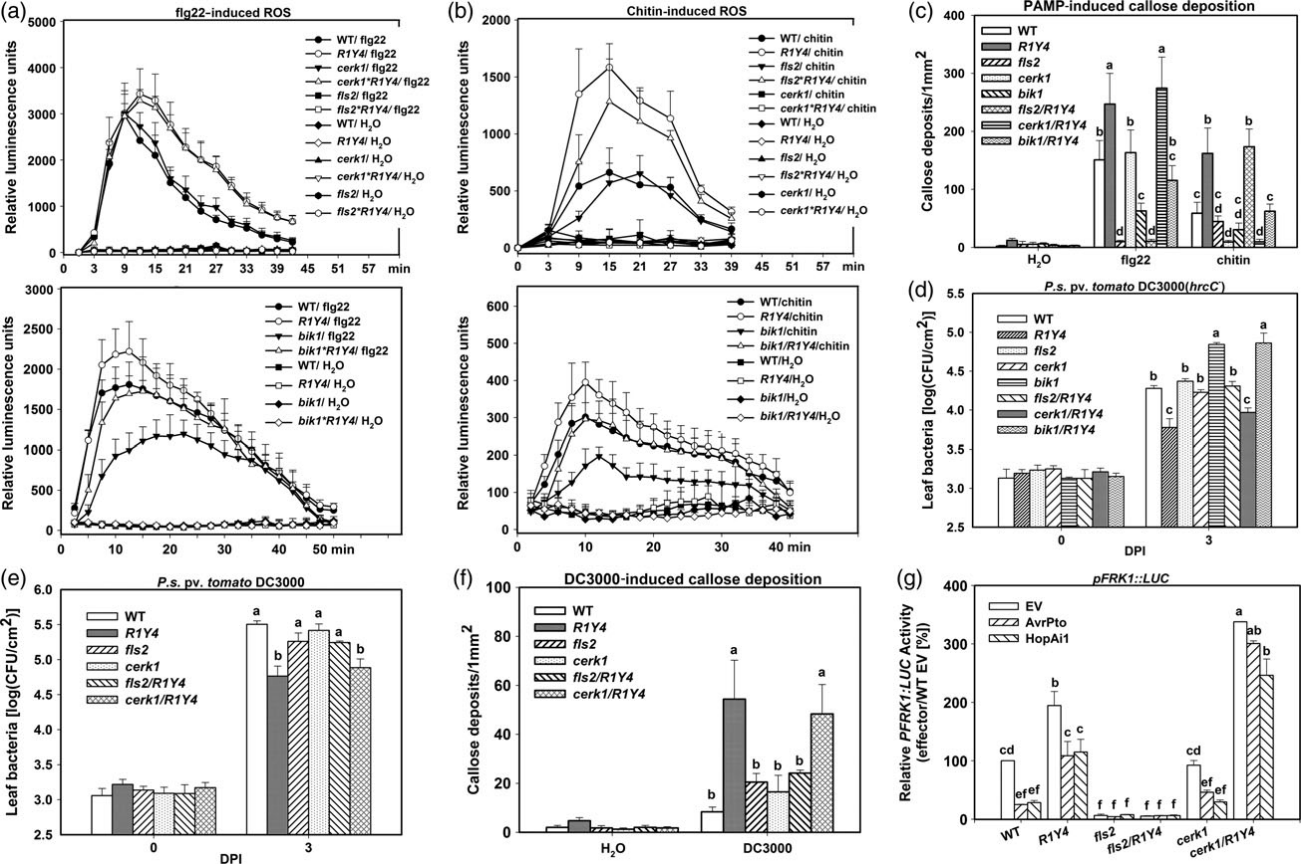
PTI signalling is required for *RPW8.1*‐enhanced defence responses. (a, b) Burst of reactive oxidative species (ROS) induced by flg22 (a) and chitin (b) in the indicated lines in comparison with the wild‐type (WT) Col‐*gl*. Error bars indicate SD (*n *= 4). (c) Quantitative analysis of PAMP‐induced callose deposition in the indicated lines. Error bars indicate standard deviation (SD,* n *= 6). (d, e) Bacterial growth assay for *P. syringae* DC3000(*hrcC* ^*‐*^) (d) and *P. syringae* DC3000 (e) in the indicated lines. Error bars indicate SD (*n *= 4). (f) *P. syringae* DC3000‐induced callose deposition in the indicated lines. Error bars indicate SD (*n *= 6). (g) Comparison of AvrPto‐ or HopAi1‐mediated suppression on flg22‐induced *FRK1::LUC* expression in the indicated lines. The *PFRK1:LUC* reporter activity (%) was normalized to the activity in WT protoplasts transfected without effectors. Error bars indicate SD (*n *= 3). Different letters above the bars in (c‐g) indicate significant differences at *P *< 0.01. All the experiments were independently repeated twice with similar results.

Next, we tested whether defects in PTI signalling can abolish or compromise *RPW8.1*‐mediated immunity. We made *fls2*/R1Y4, *cerk1*/R1Y4 and *bik1*/R1Y4 lines through crossing *fls2*,* cerk1* and *bik1* mutant with R1Y4, respectively, and found diminishment of R1Y4's pits/bulge phenotypes in these mutants’ background (Ma *et al*., 2014) (Figure S4a), although RPW8.1‐YFP was still inducible as indicated by increased YFP signal in *cerk1*/R1Y4 and *bik1*/R1Y4 upon flg22 application, and in *fls2*/R1Y4 and *bik1*/R1Y4 upon chitin application (Figure S5). While ROS burst and callose deposition induced by flg22 or chitin were completely abolished in *fls2*/R1Y4 or *cerk1*/R1Y4, respectively, flg22‐induced defence responses in *cerk1*/R1Y4 and chitin‐induced defence responses in *fls2*/R1Y4 were the same as those in R1Y4 (Figure 6a–c). ROS production and callose deposition in *bik1*/R1Y4 were compromised to the same levels as in WT, which were obviously lower than that in R1Y4 (Figure 6a–c).

Consistent with the PTI responses, multiplication of the mutant bacteria *P. syringae* DC3000(*hrcC* ^*‐*^) in *fls2*/R1Y4 and *bik1*/R1Y4 was similar to that in *fls2* and *bik1*, respectively, which was significantly higher than that in R1Y4, whereas the bacterial growth was similar in *cerk1*/R1Y4 and R1Y4 (Figure 6d). Furthermore, the multiplication of the virulent strain *P. syringae* DC3000 in *fls2*/R1Y4 was similar to that in *fls2*, which was significantly higher than that in R1Y4, whereas the growth was similar in *cerk1*/R1Y4 and R1Y4 (Figure 6e). Consistently, *P. syringae* DC3000‐induced callose deposition in *fls2*/R1Y4 decreased to the level similar to that in *fls2* plants, significantly lower than that in R1Y4 and *cerk1*/R1Y4 (Figure 6f). The expression of *FRK1* reporter in WT protoplasts was suppressed significantly by both AvrPto and HopAi1, whereas the suppression was significantly relieved in R1Y4 and *cerk1*/R1Y4 (Figure 6g). Intriguingly, the expression of *FRK1* reporter in *cerk1*/R1Y4 was significantly higher than that in R1Y4 (Figure 6g). Considering that the expression of *FRK1* is also highly inducible by senescing (Robatzek and Somssich, 2002), the higher level of *FRK1* transcription in *cerk1*/R1Y4 might have resulted from early leaf senescence. Moreover, *cerk1*/R1Y4 displayed susceptibility to a virulent powdery mildew strain, whereas *fls2*/R1Y4 showed similar resistance as seen in R1Y4 (Figure S4b).

These results indicate that PTI signalling is required for *RPW8.1*‐mediated defence responses and resistance to virulent pathogens.

### Ectopic expression of *RPW8.1* in rice enhances postinvasive resistance to *P*. *oryzae*

**Figure 7:**
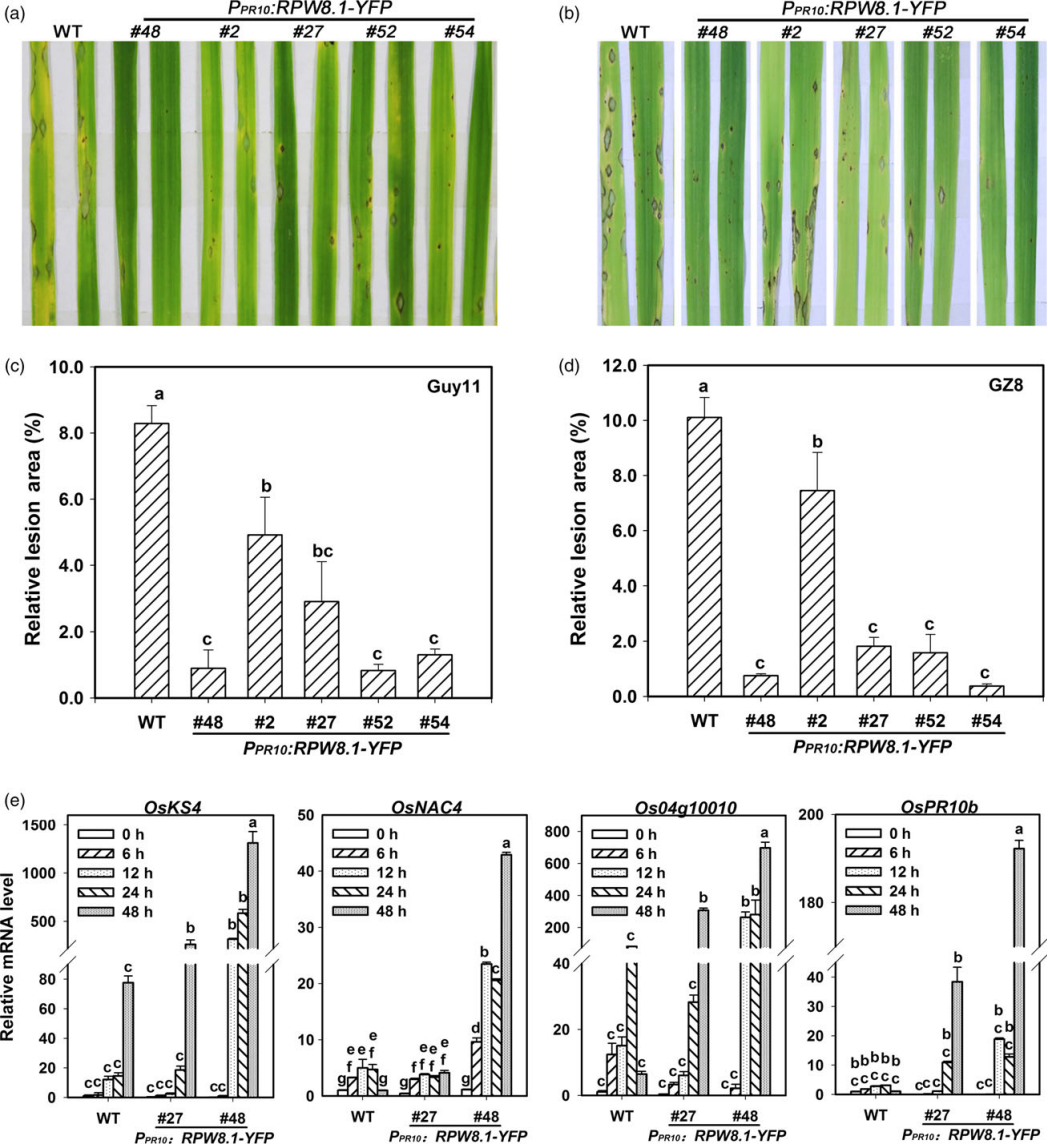
Ectopic expression of *RPW8.1‐YFP* enhances resistance against *P. oryzae* in rice. (a‐d) Representative leaf sections from the indicated transgenic lines and the wild‐type (WT) TP309 show the blast disease phenotypes (a, b) and statistical analyses on the lesion area (c, d) caused by the *P. oryzae* strain Guy11 (a, c) and the eGFP‐tagged strain GZ8 (b, d). Error bars indicate SD (*n *= 10). (e) *P. oryzae* induced expression of the indicated defence‐related genes in the indicated transgenic rice lines in comparison with WT. Relative mRNA levels were normalized to that in untreated WT plants. Error bars indicate SD (*n *= 3). Different letters above the bars in (c‐e) indicate significant differences at *P *< 0.01. All the experiments were independently repeated two times with similar results.

That RPW8.1 boosts PTI in Arabidopsis prompted us to test whether *RPW8.1* could improve resistance in rice. We thus constructed transgenic rice lines expressing *RPW8.1‐YFP* from the rice *OsPR10a* (*Os12g36830*) promoter for achieving *P*. *oryzae*‐inducible expression (Hashimoto *et al*., 2004; McGee *et al*., 2001) in TP309, a *Japonica* accession susceptible to *P*. *oryzae* and *Xanthomonas oryzae* pv. *oryzae* (*Xoo*). Upon inoculation of *P*. *oryzae*, the expression of *RPW8.1‐YFP* was increased at the transcriptional and translational level (Figure S6a,b), and the YFP signal from the fusion protein was also observed in sheath cells (Figure S6c), indicating that the *OsPR10a* promoter was indeed inducible by *P. oryzae*. The transgenic rice lines (T3) expressing *RPW8.1‐YFP* displayed enhanced resistance against the virulent *P. oryzae* strains Guy11 and eGFP‐tagged Zhong8‐10‐14 (GZ8) as evidenced by less and smaller disease lesions on inoculated leaves (Figure 7a–d). Then, two transgenic lines were used for examination of the expression of defence‐related genes, including *Kaurene Synthase4* (*OsKS4*), *OsNAC4* (for *Oryza sativa no apical meristem* [*NAM*]) (Park *et al*., 2012), *OsPR10b* and *OsMAS1* (*Os04 g10010*) (Li *et al*., 2014b; Miyamoto *et al*., 2016; Yamaguchi *et al*., 2013). The expressions of all tested genes were induced to levels remarkably higher in the transgenic line #48 than those in the control line at 12‐48 HPI, while *OsKS4* was induced to significantly higher levels at 48 HPI, and *OsPR10b* and *OsMAS1* were induced to remarkably higher levels at 24‐48 HPI in the transgenic line #27 (Figure 7e).

Next, we detected induction of *RPW8.1‐YFP* and the defence‐related genes *OsNAC4* and *OsKS4* in the transgenic rice lines by PAMPs. The relative mRNA level of *RPW8.1* was up‐regulated at 6 HPI of chitin and at 12 HPI of flg22 (Figure S7a). The RPW8.1‐YFP protein levels were up‐regulated in the transgenic lines upon flg22‐ or chitin application (Figure S7b), and the YFP signals were also increased in the sheath cells (Figure S7c). In addition, both *OsKS4* and *OsNAC4* were induced to obviously higher levels in the transgenic lines than that in the control line upon flg22 or chitin application (Figure S7d,e).

**Figure 8:**
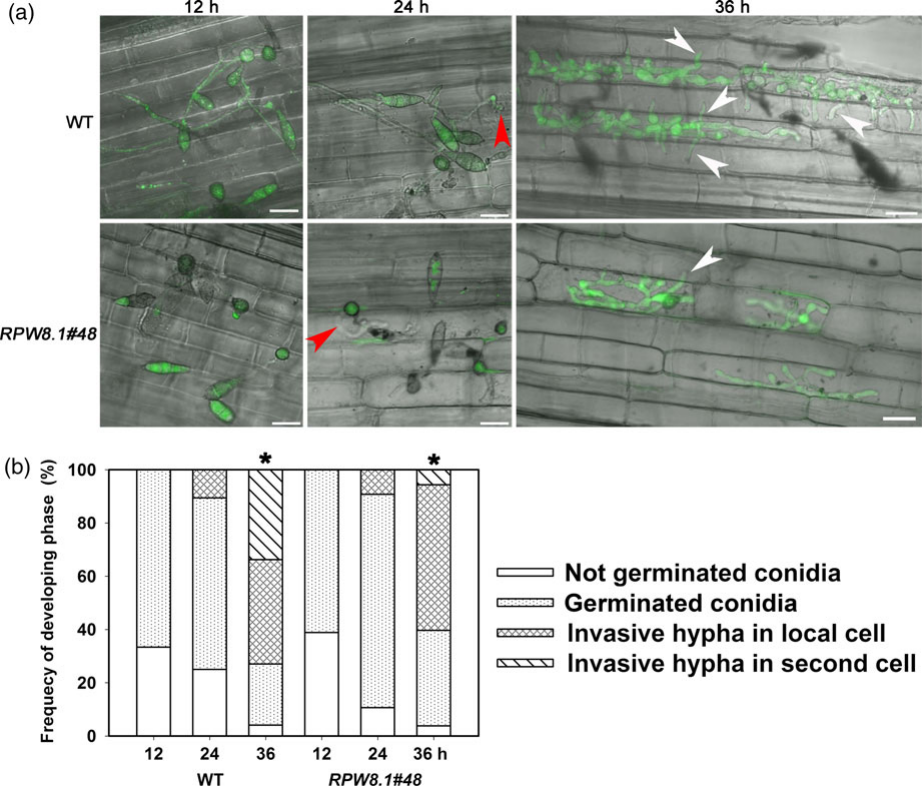
Ectopic expression of *RPW8.1‐YFP* in rice enhances postinvasive resistance to *P. oryzae*. (a) Confocal images show the infection process of the eGFP‐tagged strain Zhong‐8‐10‐14 on sheath cells from the wild‐type (WT) TP309 and the transgenic line expressing *RPW8.1‐YFP*. Note that appressoria (arrows) were observed at 12 h postinoculation (HPI). Invasive hyphae (red arrowheads) in the primary infected cells were observed at 24 HPI. The invasive hyphae extended to the neighbour cells (white arrowheads) at 36 HPI. Size bars, 20 μm. Similar results were obtained in two independent experiments. (b) Quantitative analyses on the process of infection from at least 50 conidia at the indicated time points on the indicated lines. Note that the number of invasive hyphae extended from the primary infected cells into the neighbouring cells is obviously lower in the *RPW8.1‐YFP* transgenic line than that in the control WT at 36 HPI (*).

We also observed less aggression of GZ8 on leaf sheath from one *RPW8.1* transgenic rice line compared with the control line. The GZ8 spores germinated at 12 HPI, and the percentage of germinated spores did not display significant difference (Figure 8). The primary invasive hyphae were formed at 24 HPI, and the invasive hyphae extended from the primary infected cells to the neighbouring cells at 36 HPI (Figure 8a). Notably, the percentage of the hyphae extending into the neighbour cells in the transgenic line expressing *RPW8.1‐YFP* was significantly lower than that in WT (Figure 8b).

These observations indicate that ectopic expression of *RPW8.1‐YFP* can also enhance PTI in rice to render postinvasive resistance against *P. oryzae*

### Ectopic expression of *RPW8.1* in rice results in enhanced resistance to *Xoo*

**Figure 9:**
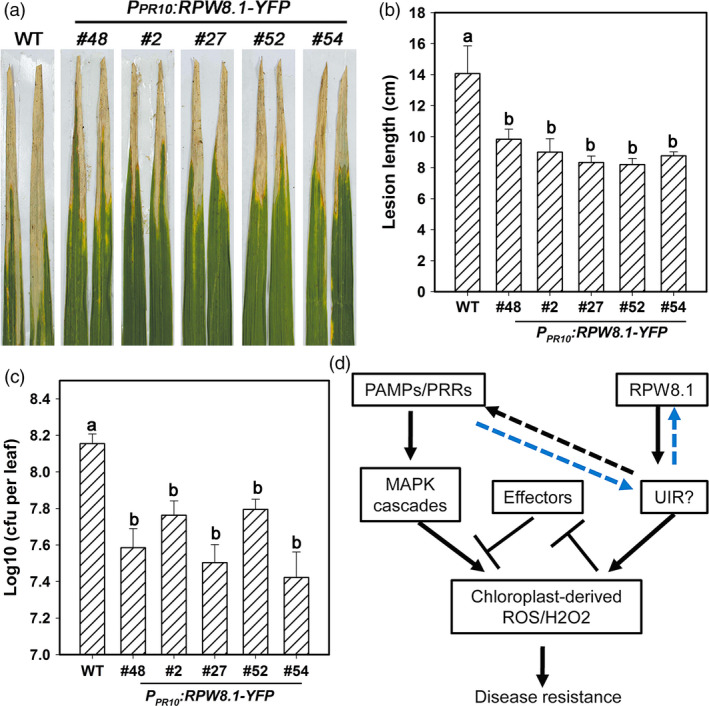
Ectopic expression of *RPW8.1‐YFP* in rice enhances resistance to *Xoo*. (a) Leaf sections from the indicated transgenic lines show the disease phenotypes in comparison with the wild‐type (WT) TP309 at 14 days postinoculation (dpi) of *Xoo*. Similar results were obtained in two independent experiments. (b, c) Statistical analysis on lesion length (b) and bacterial growth (c) at 14 dpi. Error bars indicate SD (*n *= 10). Different letters above the bars indicate significant differences at *P *< 0.01. All the experiments were independently repeated twice with similar results. (d) A hypothetical model for the RPW8.1‐PTI connection.

That expression of *RPW8.1‐YFP* was inducible by flg22 in transgenic rice prompted us to test whether the *RPW8.1* transgenic rice lines are resistant to the bacterial pathogen *Xoo*, the causative agent of rice leaf blight disease. As shown in Figure 9a, all of the *RPW8.1‐YFP* transgenic lines displayed alleviated symptom and shorter lesions compared to the control plants at 14 dpi of POX99. The average lesion length of the transgenic lines was in the range of 8 to 10 cm at 14 dpi, significantly shorter than that (~15 cm) of control plants (Figure 9b). Quantitative analysis revealed that the transgenic lines expressing *RPW8.1‐YFP* supported significantly less bacterial growth than the control plants (Figure 9c). These data indicate that ectopic expression of *RPW8.1* in rice results in enhanced resistance to *Xoo*.

However, we also noticed that ectopic expression of *RPW8.1‐YFP* in rice led to some negative impact on agronomic traits. As shown in Table S4, the yield‐component traits, including number of filled grain per panicle and 1000‐grain weight, were significantly lower in the transgenic lines than in the WT, indicating substantial fitness penalties, despite we used the pathogen‐inducible promoter of *OsPR10a*. The recently reported uORF‐mediated translational control system (Xu *et al*., 2017) may be used to reduce the cost of resistance associated with *R* genes such as *RPW8.1* when they are exploited for engineering disease resistance in crops in the future.

## Discussion

Previously, we found that ectopic expression of *RPW8.1* activates resistance to virulent powdery mildew and oomycete pathogens (Ma *et al*., 2014). Here, we further found that ectopic expression of *RPW8.1* also enhances resistance against a virulent bacterial strain (Figure 1), and provided evidence that *RPW8.1*, but not *RPW8.2*, boosts PTI basal defence signalling to activate defence responses against different virulent pathogens. First, higher PTI responses such as ROS burst, callose deposition and defence gene expression were consistently observed in R1Y4 but not in R2Y4 upon PAMP application and bacterial infection, although both *RPW8.1* and *RPW8.2* seemed to be inducible by PAMPs (Figure S1) and bacteria (Figure S3). Second, the defence responses triggered by virulent powdery mildew and oomycete pathogens observed in a previous report (Ma *et al*., 2014), such as H_2_O_2_ accumulation and cell death, and the transcription of *PR* genes, were all induced by PAMP application in R1Y4 but not in R2Y4 (Figure 4). Third, the ectopic expression of *RPW8.1* constitutively up‐regulated many PAMP‐inducible genes in R1Y4, and over 50% of the *RPW8.1*‐up‐regulated genes were consensually inducible by flg22 and chitin in WT plants (Figure 2). Fourth, the PAMP‐triggered transcription of *PRR*s and their downstream PTI components were significantly increased in *RPW8.1*‐transgenic plants (Figure S2). Conversely, *RPW8.1*‐mediated up‐regulation of flg22‐induced and chitin‐induced PTI responses were completely abolished in the *fls2* or *cerk1* background, respectively, and significantly compromised in the *bik1* background (Figure 6a–d). Consequently, *RPW8.1*‐mediated PTI responses and resistance to the virulent bacterial pathogen were also completely abolished in *fls2* mutant background (Figure 6e–g). Taken together, these results indicate that *RPW8.1* and *RPW8.2* activate different resistance mechanisms to mount defences against pathogen invasion, and PTI signalling is required for *RPW8.1*‐mediated up‐regulation of defence responses against different virulent pathogens.

During plant–microbe co‐evolution, adapted pathogens use effectors to subvert PTI. In turn, plant R proteins recognize directly or indirectly cognate effectors to mount ETI that usually culminates in HR (Jones and Dangl, 2006). Even though it is unlikely that RPW8.1 plays a role in effector recognition, in this study we did observe ETI‐like defence responses in plants expressing RPW8.1‐YFP upon infection of a virulent bacterial strain (Figures 1 and 5). A plausible explanation is that expression of RPW8.1 can significantly boost PTI, thereby counterbalancing the suppression of PTI by pathogen effectors. More intriguingly, we observed attenuation of both effector secretion and effector virulence as a result of ectopic expression of *RPW8.1* (Figure 5e–g). How expression of RPW8.1 could achieve this is currently unknown. The distinct subcellular localization of RPW8.1‐YFP may provide a clue. First, RPW8.1‐YFP discretely accumulated around chloroplasts in mesophyll cells, which may cause stress on chloroplasts, leading to constitutive defence responses (Figure 2 and Ma *et al*., 2014). Second, upon PAMP application or pathogen infection, RPW8.1‐YFP expressed from the *RPW8.1* promoter was further up‐regulated and formed big fluorescent punctate bodies proximal to chloroplasts in Arabidopsis mesophyll cells. Enhanced RPW8.1 expression likely heightened PTI signalling and defence responses via a feedback amplification circuit (Figures 3, 4, S2 and S3), leading to H_2_O_2_ production and cell contraction before the collapse of chloroplasts in the early infection period. During the late infection period, chloroplasts in the H_2_O_2_‐enriched cells disintegrated followed by cell demise, which further resulted in cytoplasmic bubbling in neighbour cells (Figure 4d). In turn, it is conceivable that the heightened defence responses, such as chloroplast‐produced ROS/H_2_O_2_, in RPW8.1‐expressing Arabidopsis leaf cells might inhibit the secretion of virulent effectors and/or counterbalance the virulent effectors’ suppression of PTI. Consistently, in transgenic rice plants, most of the RPW8.1‐YFP fluorescent punctate bodies were also found to be localized proximal to the chloroplasts/plastids upon *P. oryzae* infection (Figure S6c) and PAMP treatment (Figure S7c). Similar PTI‐boosting mechanism may be activated by expression of RPW8.1 in rice cells, resulting in reduced infection of *P. oryzae* and *Xoo*. These observations on chloroplast/plastid‐associated localization of RPW8.1‐YFP in both Arabidopsis and rice point a likely role of chloroplasts/plastids for *RPW8.1*‐mediated basal resistance against virulent pathogens with possibly attenuation of effector secretion and/or their virulence activities.

Based on the results from this work and our previous studies, we hypothesize that RPW8.1 might be associated with an unidentified immune regulator (UIR) (Figure 9d). Ectopic expression of *RPW8.1* may activate this hypothetical UIR that in turn up‐regulates basal chloroplast‐derived ROS/H_2_O_2_, thereby activating defence responses. Upon PAMP recognition, MAPK cascades are activated, resulting in further up‐regulation of chloroplast‐derived ROS/H_2_O_2_. During this process, the PTI signalling may modulate the UIR that may further up‐regulate RPW8.1 via a positive feedback circuit. In turn, increased expression of RPW8.1 could further enhance PTI signalling through increased expression or recruitment of PRRs and strengthening of the MAPK cascades, possibly via the UIR, leading to inhibition of effector‐mediated virulence, increased production of chloroplast‐derived ROS/H_2_O_2_ and other defence responses. Although the intrinsic mechanistic connection between RPW8.1 and PTI via the UIR remains to be fully characterized, this regulatory node may be conserved between dicots and monocots, making *RPW8.1* a promising *R* gene for engineering disease resistance across broad taxonomic lineages of crop species.

## Experimental procedures

### Generation of transgenic plants

Arabidopsis accessions Shahdara, Wa, Ws and transgenic lines expressing RPW8.1‐YFP (i.e. R1Y2,R1Y4 and R1Y5) and expressing RPW8.2‐YFP (i.e. R2Y4) in the WT Col‐*gl* background were from previous reports (Ma *et al*., 2014; Orgil *et al*., 2007; Wang *et al*., 2009). To generate ectopic expression of RPW8.1‐YFP in PTI signalling mutant background, R1Y4 was crossed with *fls2* (SALK_141277), *cerk1* (SALK_092023) and *bik1* (CS852520) (Veronese *et al*., 2006; Wan *et al*., 2008; Xiang *et al*., 2008). Homologous *fls2, cerk1* and *bik1* in *fls2*/R1Y4, *cerk1*/R1Y4 and *bik1*/R1Y4 were obtained by screening F_2_ individuals with gene‐specific primers fls2‐F/fls2‐R, cerk1‐F/cerk1‐R, bik1‐F/bik1‐R and T‐DNA primer LB (Table S3). Arabidopsis plants were grown in a growth room maintained at 23 °C and 70% relative humidity with a 10‐/14‐h day/night regime.

To generate transgenic rice lines expressing *RPW8.1‐YFP*, the cassette *RPW8.1‐YFP* in the plasmid pPR81EYFP from Wang *et al*. (2007) was put downstream to the *OsPR10a* (*Os12 g36830*) promoter that was amplified with POsPR10‐F and POsPR10‐R (Table S3), leading to the construct pP_PR10_:RPW8.1‐YFP. Then, the construct pP_PR10_:RPW8.1‐YFP was introduced into rice accession TP309 following a previous report (Li *et al*., 2014b). Rice plants were grown either in paddy fields or in a growth room maintained at 26 °C and 70% relative humidity with a 14‐/10‐h day/night regime.

### Bacterial disease assay in Arabidopsis

Five‐week‐old Arabidopsis plants were syringe‐infiltrated with the virulent strain *P. syringae* DC3000 at the concentration of OD_600_ = 0.0005, the avirulent strains *P. syringae* DC3000(*avrRpm1*) and DC3000(*avrRpt2*) at OD_600_ = 0.002, or spray‐inoculated with the mutant strain *P. syringae* DC3000(*hrcC* ^*‐*^) at OD_600_ = 0.5. (Yuan and He, 1996). Bacterial propagation was determined as previously described (Li *et al*., 2010) at 0 and 3 dpi, respectively.

### RNA‐Seq analysis

Five‐week‐old plants of Col‐*gl* and R1Y4 were infiltrated with 1 μm of flg22 or 20 μg/mL of chitin, and samples were collected at 0, 1, 3, 6 and 12 HPI. Total RNA was extracted by TRIzol Reagent (Invitrogen, Thermo Fisher Scientific, Shanghai, China). RNA quality was determined using Agilent 2100 Bioanalyzer (Agilent Technologies Canada Inc, Mississauga, ON, Canada). RNA libraries were constructed from 2 μg of total RNA and subjected to deep sequencing at an Illumina Hiseq 2500 platform (BioMarker Technologies Illumina, Inc, Shanghai, China). After removing adaptor sequences and filtering low‐quality sequences, clean reads from each samples were mapped to the TAIR10 reference genome by TopHat2 with default parameters (Kim *et al*., 2013). The FPKM (fragments per kilobase of transcript per million fragments mapped) method was used to calculate the normalized expression data of each library (Mortazavi *et al*., 2008). Differentially expressed genes (DEGs) were identified by DEseq2 (Love *et al*., 2014) with the criteria of absolute log2 (fold change) ≥1 and false discovery rate (FDR) ≤0.01. Gene ontology (GO) enrichment analysis on DEGs was performed using GOseq (Young *et al*., 2010).

### Assays for PTI defence responses in Arabidopsis

For examining the activation of MAPKs, 5‐week‐old plants were sprayed with 10 μm of flg22 in 0.02% Silwet L‐77 and samples were collected for protein extraction at 5 and 10 min after PAMP application. Fifteen micrograms of total protein was electrophoresed on 10% SDS‐PAGE gel, and the protein blot was reacted with anti‐p‐ERK serum (Cell Signaling Technology, Danvers, USA) to detect and determine phosphorylation status of MPK3, MPK4 and MPK6 as previously described (Li *et al*., 2010). For examining the production of ROS, leaf strips were incubated in 200 μL water in a 96‐well plate for 12 h, and treated with 1 μm flg22 or 20 μg/mL chitin in 200 μL buffer containing 20 mm luminol, 10 μg/mL horseradish peroxidase (Sigma‐Aldrich Shanghai Trading Co Ltd, Shanghai, China). ROS burst indicated as relative luminescence units was determined with a GLOMAX96 Microplate Luminometer (Promega (Beijing) Biotech Co., Ltd, Beijing, China) for 40–60 min. For examining callose deposition, leaves syringe‐infiltrated with 1 μm flg22 or 1 20 μg/mL chitin, or *P. syringae* DC3000 (OD_600_ = 0.002) were collected, cleared, and stained with 0.01% aniline blue for half an hour following a previous report (Hauck *et al*., 2003). Representative images of callose deposition were captured with a fluorescence microscope (Zeiss imager A2.0) and calculated using ImageJ as previously reported (Zhang *et al*., 2007).

### Quantitative RT‐PCR, Western blot and Microscopy analysis

Quantitative RT‐PCR was performed following previous reports (Li *et al*., 2010) with *Actin2* (in Arabidopsis) or *OsUbi1* (in rice) as internal control. Statistical analysis was performed by t‐test or by a one‐way ANOVA followed by post hoc Tukey HSD analysis. For Western blot analysis, protein extraction and gel blotting were performed as described in a previous report (Wang *et al*., 2007) with antigreen fluorescent protein (GFP) serum to detect YFP‐tagged protein. Accumulation of YFP‐tagged proteins were observed, and images were acquired by LSCM as previously described (Huang *et al*., 2014) using Nikon80i. H_2_O_2_ production and dead cells were stained by DAB and trypan blue, respectively (Xiao *et al*., 2003).

### Reporter assay in protoplasts

Protoplasts isolated from 5‐week‐old plants of Col‐*gl*, R1Y4, R2Y4, *fls2*,* fls2*/R1Y4, *cerk1*,* cerk1*/R1Y4 were cotransfected with *FRK1::LUC* (firefly luciferase) and *35S::RLUC* (R*LUC*, Renilla luciferase) along with the *35S::AvrPto* or *35S::HopAi1* or empty vector (EV) constructs as described (Zhang *et al*., 2010). Proteins were isolated with the Dual‐Luciferase Reporter system kit (Promega) following the manufacturer's instructions, and LUC activity was determined with the GLOMAX96 Microplate Luminometer (Promega). The reads of R*LUC* were used as internal control for the reads of *FRK1* reporter. The relative expression level of *FRK1* reporter in *35S::AvrPto‐* or *35S::HopAi1*‐cotransfected samples was normalized to those in *EV*‐cotransfected samples.

### Adenylate cyclase assay

To assay the secretion of bacterial effectors in plant tissue, leaves from 5‐week‐old Arabidopsis plants were syringe‐infiltrated with strain *P. syringae* DC3000(AvrPto‐Cya) (OD_600_ = 0.05). Then, treated leaves were collected at 0, 6, 9 and 12 HPI. Half of the treated leaves were used to measure the bacterial propagation, and half were used to measure the activity of adenylate cyclase as previously described (Schechter *et al*., 2004).

### *P. oryzae* inoculation and disease resistance assay

The *P. oryzae* strains Guy11 and GZ8 were grown and inoculated as previously described (Li *et al*., 2014b). For monitoring the infection process of *P. oryzae* to rice, the spore suspensions of GZ8 were inoculated on 10‐cm‐long leaf sheaths and the inoculated epidermal layer was excised and analysed as described (Kankanala *et al*., 2007). Images were acquired using LSCM (Huang *et al*., 2014)

### *Xoo* inoculation and bacterial leaf blight disease assay

Six‐week‐old rice plants were cut‐inoculated with the *Xoo* strain PXO99, and the disease lesion length and bacterial growth in the inoculated leaves were determined according to a previous report (Chern *et al*., 2005).

## Conflict of interest

The authors declare no conflict of interest.

## Supporting information


**Figure S1** PAMPs up‐regulate the expression of *RPW8.1* and *RPW8.2*.
**Figure S2** Ectopic expression of RPW8.1‐YFP enhances the transcription of *PRR*s and PTI components upon application of PAMPs.
**Figure S3** *P. syringae* DC3000 up‐regulates the expression of *RPW8.1‐YFP* and *RPW8.2‐YFP*.
**Figure S4** PTI signaling is required for *RPW8.1*‐mediated resistance to powdery mildew in Arabidopsis.
**Figure S5** PTI signaling is required for PAMP‐induced accumulation of RPW8.1‐YFP. Representative confocal images show the subcellular accumulation of PAMP‐induced RPW8.1‐YFP in the indicated lines.
**Figure S6** *P. oryzae* up‐regulates the expression of *RPW8.1* in transgenic rice plants.
**Figure S7** PAMPs up‐regulate expression of *RPW8.1* in transgenic rice plants.

**Table S1** List of the 309 flg22‐/chitin‐inducible and RPW8.1‐up‐regulated genes.

**Table S2** GO term enrichment of the 309 flg22‐/chitin‐inducible and RPW8.1‐ up‐regulated genes.

**Table S3** Primers used in this study.

**Table S4** Comparison of some agronomic traits between wild‐type and transgenic lines.
