# Supplementary material for: RESISTANCE TO POWDERY MILDEW8.1 boosts pattern‐triggered immunity against multiple pathogens in Arabidopsis and rice
Source: Plant Biotechnol J. 2017 Jul 27;16(2):428–41. doi: 10.1111/pbi.12782 (PMC5787827; doi:10.1111/pbi.12782)
Supplement: Supplementary file 1 — Figure S1 PAMPs up‐regulate the expression of RPW8.1 and RPW8.2. Figure S2 Ectopic expression of RPW8.1‐YFP enhances the transcription of PRRs and PTI components upon application of PAMPs. Figure S3 P. syringae DC3000 up‐regulates the expression of RPW8.1‐YFP and RPW8.2‐YFP. Figure S4 PTI signaling is required for RPW8.1‐mediated resistance to powdery mildew in Arabidopsis. Figure S5 PTI signaling is required for PAMP‐induced accumulation of RPW8.1‐YFP. Representative confocal images show the subcellular accumulation of PAMP‐induced RPW8.1‐YFP in the indicated lines. Figure S6 P. oryzae up‐regulates the expression of RPW8.1 in transgenic rice plants. Figure S7 PAMPs up‐regulate expression of RPW8.1 in transgenic rice plants. [file PBI-16-428-s004.pdf]

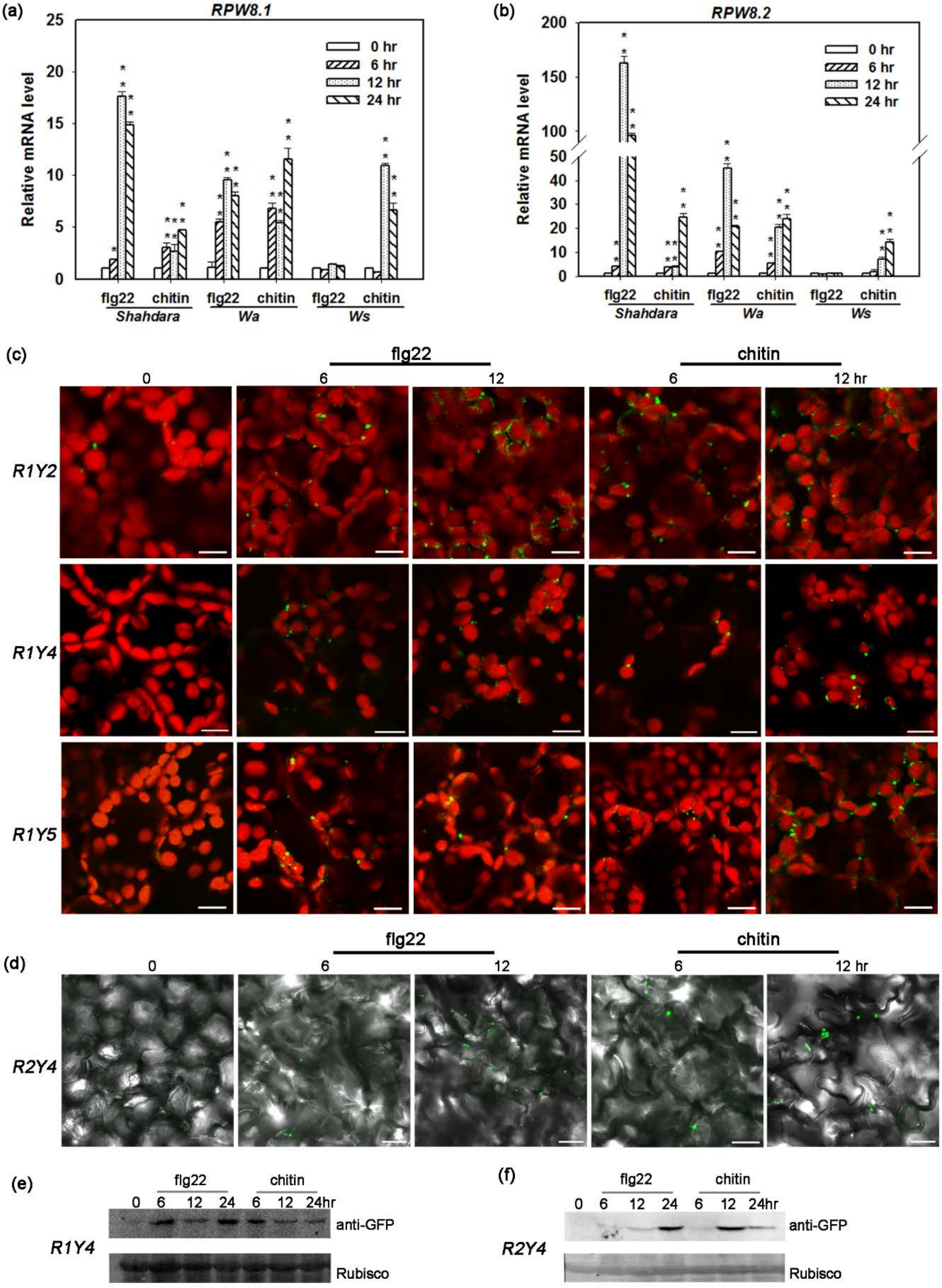

(a)
RPW8.1
Relative mRNA level
0 hr
6 hr
12 hr
24 hr
flg22
chitin
Shahdara
Wa
Ws
(b)
RPW8.2
(c)
flg22
chitin
0
6
12
12 hr
R1Y2
R1Y4
R1Y5
(d)
R2Y4
(e)
R1Y4
24hr
anti-GFP
Rubisco
(f)
R2Y4

Figure S1 PAMPs up-regulate the expression of *RPW8.1* and *RPW8.2*. (a, b) Expression of *RPW8.1* (a) and *RPW8.2* (b) in the indicated Arabidopsis accessions upon flg22 or chitin infection. Leaves from five-week-old plants were syringe-infiltrated with flg22 or chitin, and total RNA was extracted at the indicated time points for quantitative RT-PCR analysis. Relative mRNA level was normalized to that in untreated plants at 0 hour post infiltration (hpi). Error bars indicate SD (n=3). Student's *t* test was carried out to determine the significance of difference between PAMP-treated sample and untreated sample. Asterisks(**) indicated significant difference at a P value≤0.01. The experiments were repeated two times with similar results. (c, d) Representative confocal images show the subcellular accumulation of RPW8.1-YFP (c) and RPW8.2-YFP (d) in the indicated transgenic lines upon the indicated PAMP treatments at the indicated time points. The indicated transgenic lines were syringe-infiltrated with flg22 or chitin, and the infiltrated leaves were observed to acquire images under laser scanning confocal microscopy (LSCM) at the indicated time points. RPW8.1-YFP and RPW8.2-YFP were pseudo-colored green, and the auto-fluorescence from chloroplast was pseudo-colored red. Size bar, 10 μm. (e, f) Western blot analysis shows the accumulation of RPW8.1-YFP (e) and RPW8.2-YFP (f) in the indicated transgenic lines upon flg22 and chitin application. Leaves from five-week-old R1Y4 and R2Y4 plants were syringe-infiltrated with flg22 or chitin, and protein was extracted at the indicated time points for western blot analysis with anti-GFP serum. Rubisco was stained with Ponceau S as loading control.

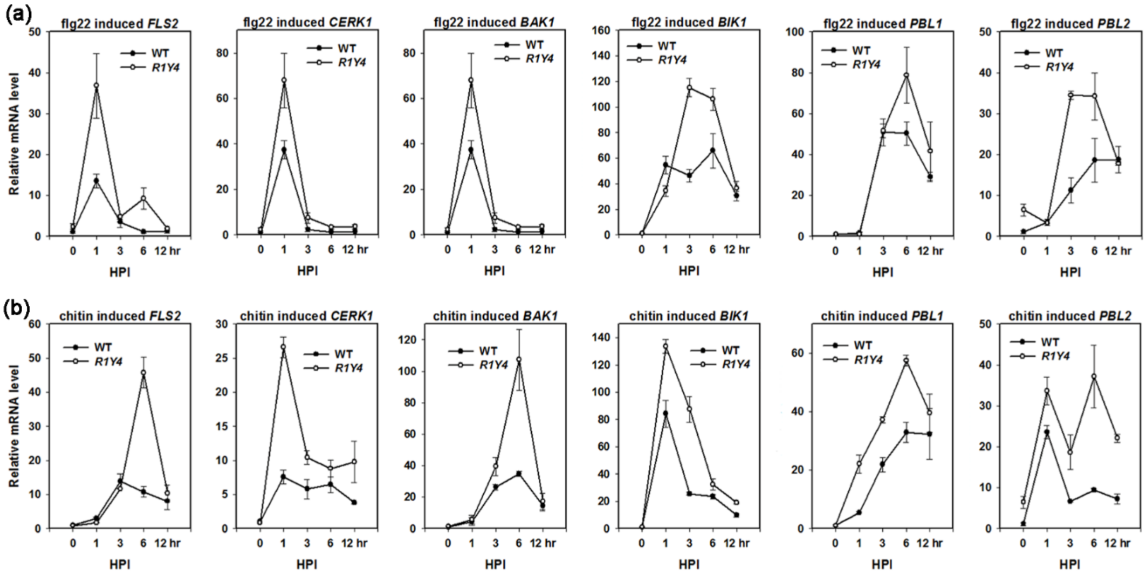

Figure S2 Ectopic expression of RPW8.1-YFP enhances the transcription of *PRR*s and PTI components upon application of PAMPs. (a, b) Expression patterns of the indicated genes in wild type (WT) Col-*gl* and the transgenic line R1Y4 upon PAMP application. Leaves from 5-week-old plants of WT and R1Y4 were syringe-infiltrated with PAMPs, and RNA was extracted at the indicated time points for quantitative RT-PCR analysis. Relative mRNA levels were normalized to that in untreated WT plants. Error bars indicate standard deviation (n=3). HPI, hours post infiltration. Similar results were obtained in two independent experiments.

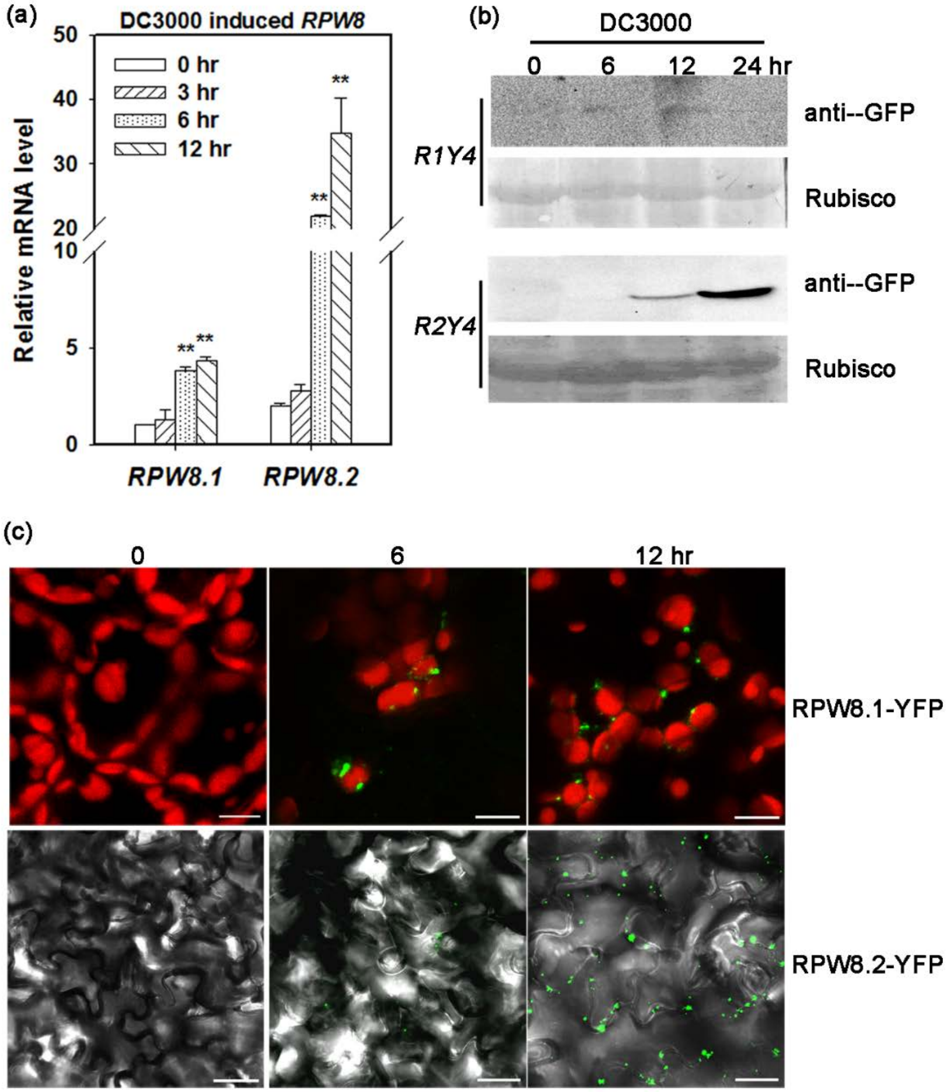

Figure S3 *P. syringae* DC3000 up-regulates the expression of *RPW8.1-YFP* and *RPW8.2-YFP*. (a) Quantitative RT-PCR data show the expression pattern of *RPW8.1* in R1Y4 and *RPW8.2* in R2Y4 upon *P. syringae* DC3000 infection. Leaves of five-week-old plants were syringe-infiltrated with *P. syringae* DC3000, and total RNA was extracted at the indicated time points for quantitative RT-PCR analysis. Relative mRNA level was normalized to that in untreated plants (0 hr). Error bars indicate SD (n=3). Student's *t* test was carried out to determine the significance of difference between PAMP-treated samples and untreated samples. Asterisks (**) indicate significant difference at a P value≤0.01. Similar results were obtained in two independent experiments. (b) Western blot analysis shows the accumulation of RPW8.1-YFP in R1Y4 and RPW8.2-YFP in R2Y4 upon *P. syringae* DC3000 infection. Five-week-old R1Y4 and R2Y4 plants were syringe-infiltrated with *P. syringae* DC3000, and protein was extracted at the indicated time points for western blot analysis with anti-GFP sera. Rubisco was stained with Ponceau S as loading control. (c) Representative confocal images show the subcellular accumulation of RPW8.1-YFP in R1Y4 and RPW8.2-YFP in R2Y4 upon *P. syringae* DC3000 infection. Five-week-old R1Y4 and R2Y4 plants were syringe-infiltrated with *P. syringae* DC3000. The infiltrated leaves were sampled to observe the subcellular accumulation of RPW8.1-YFP and RPW8.2-YFP under a laser scanning confocal microscopy (LSCM). Images were acquired at the indicated time points. RPW8.1-YFP and RPW8.2-YFP were pseudo-colored green, and the auto-fluorescence from chloroplast was pseudo-colored red. Size bar, 10 μm.

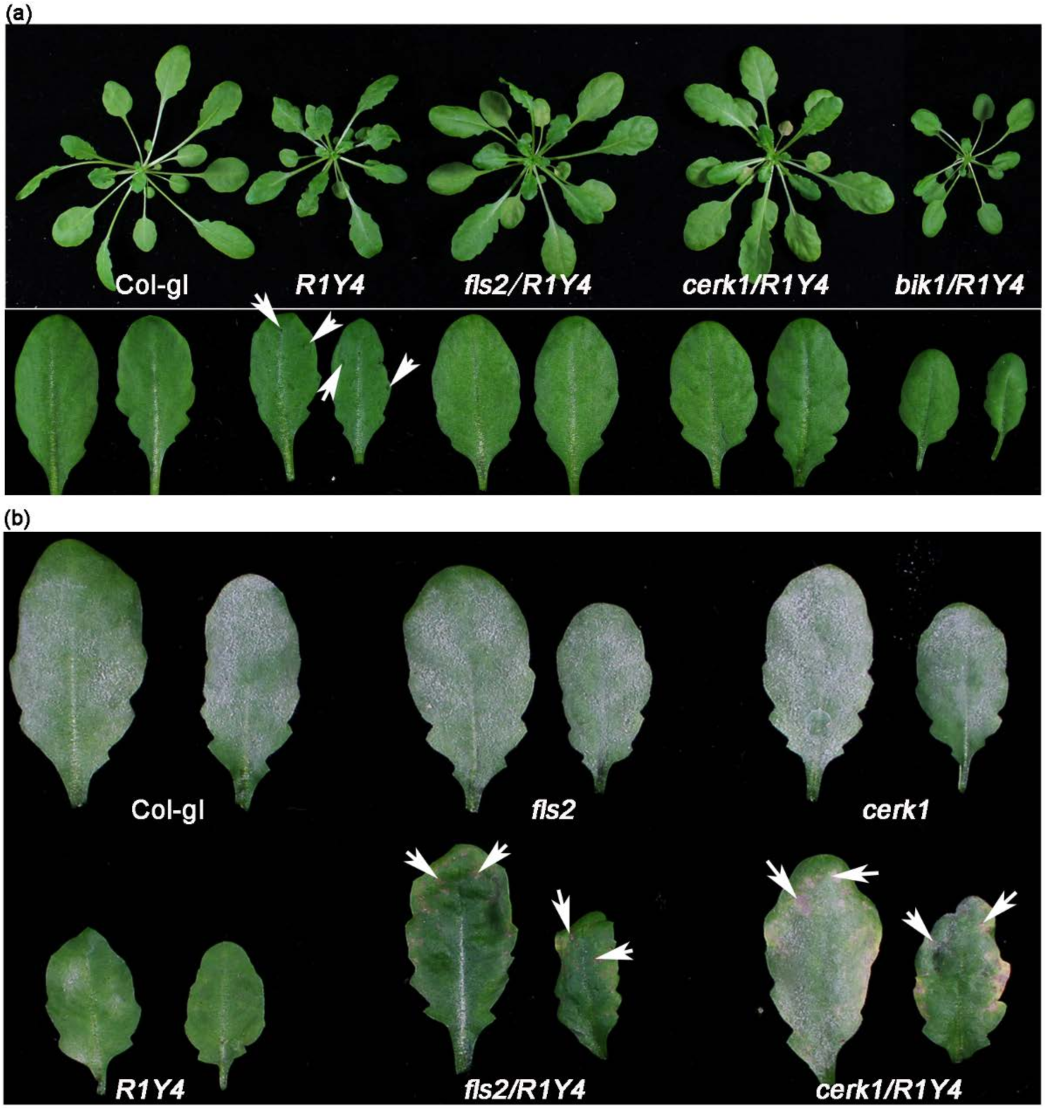

Figure S4 PTI signaling is required for RPW8.1-mediated resistance against powdery mildew in Arabidopsis. (a) Representative 6-week-old plants and leaves from the indicated lines show the pit/bulge (arrows) phenotypes caused by *RPW8.1*-mediated cell death. Note that the pit/bulge phenotypes as those in R1Y4 were abolished or alleviated in *fls2*, *cerk1* and *bik1* mutant background. (b) Representative leaves from the indicated lines show powdery mildew disease phenotypes at 8 days post inoculation. Note that *cerk1*/R1Y4 was as susceptible as Col-gl, whereas, *fls2*/R1Y4 was as resistant as R1Y4, although lesions (arrows) were observed in both *fls2*/R1Y4 and *cerk1*/R1Y4.

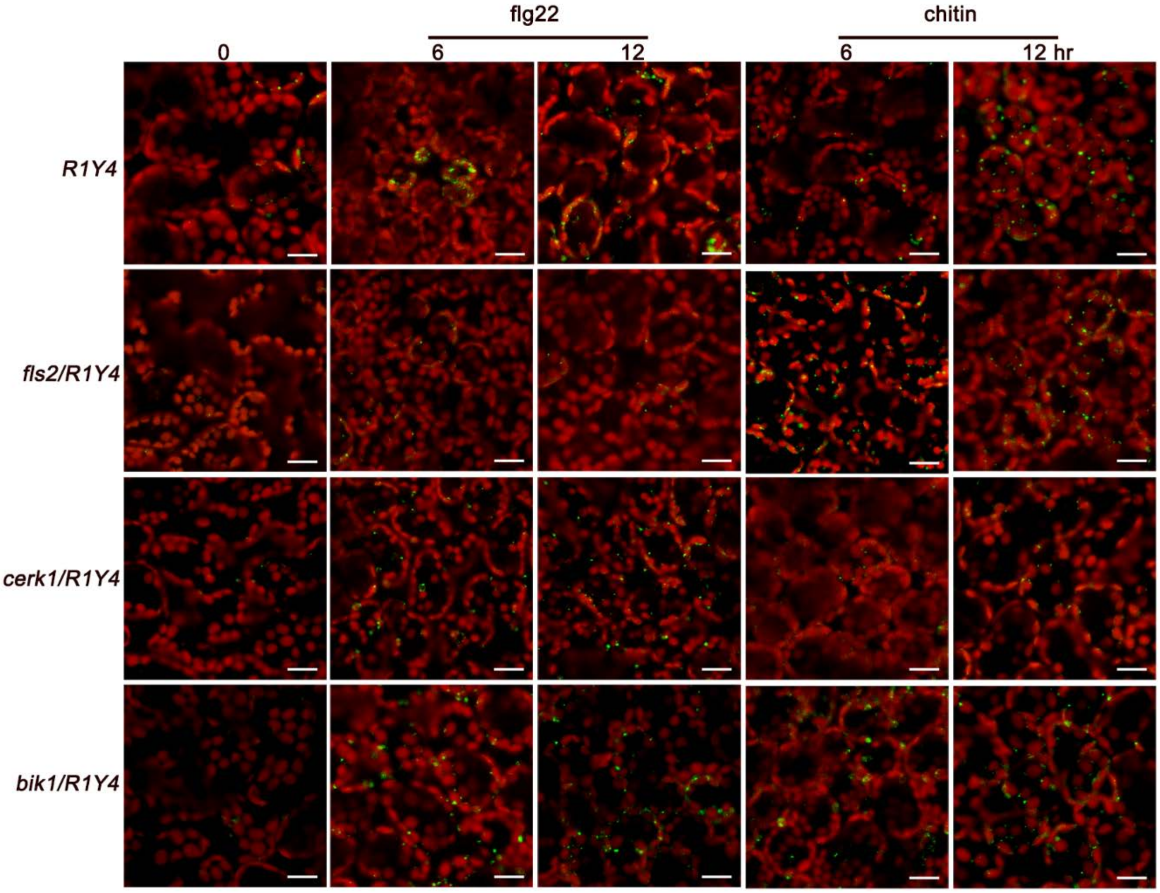

Figure S5 PTI signaling is required for PAMP-induced expression of *RPW8.1-YFP*. Representative images show the subcellular accumulation of PAMP-induced RPW8.1-YFP in the indicated lines. Five-week-old plants were syringe-infiltrated with flg22 or chitin, and images were acquired by laser scanning confocal microscopy (LSCM) at the indicated time points. RPW8.1-YFP was pseudo-colored green, and the auto-fluorescence from chloroplast was pseudo-colored red. Size bar, 10 μm.

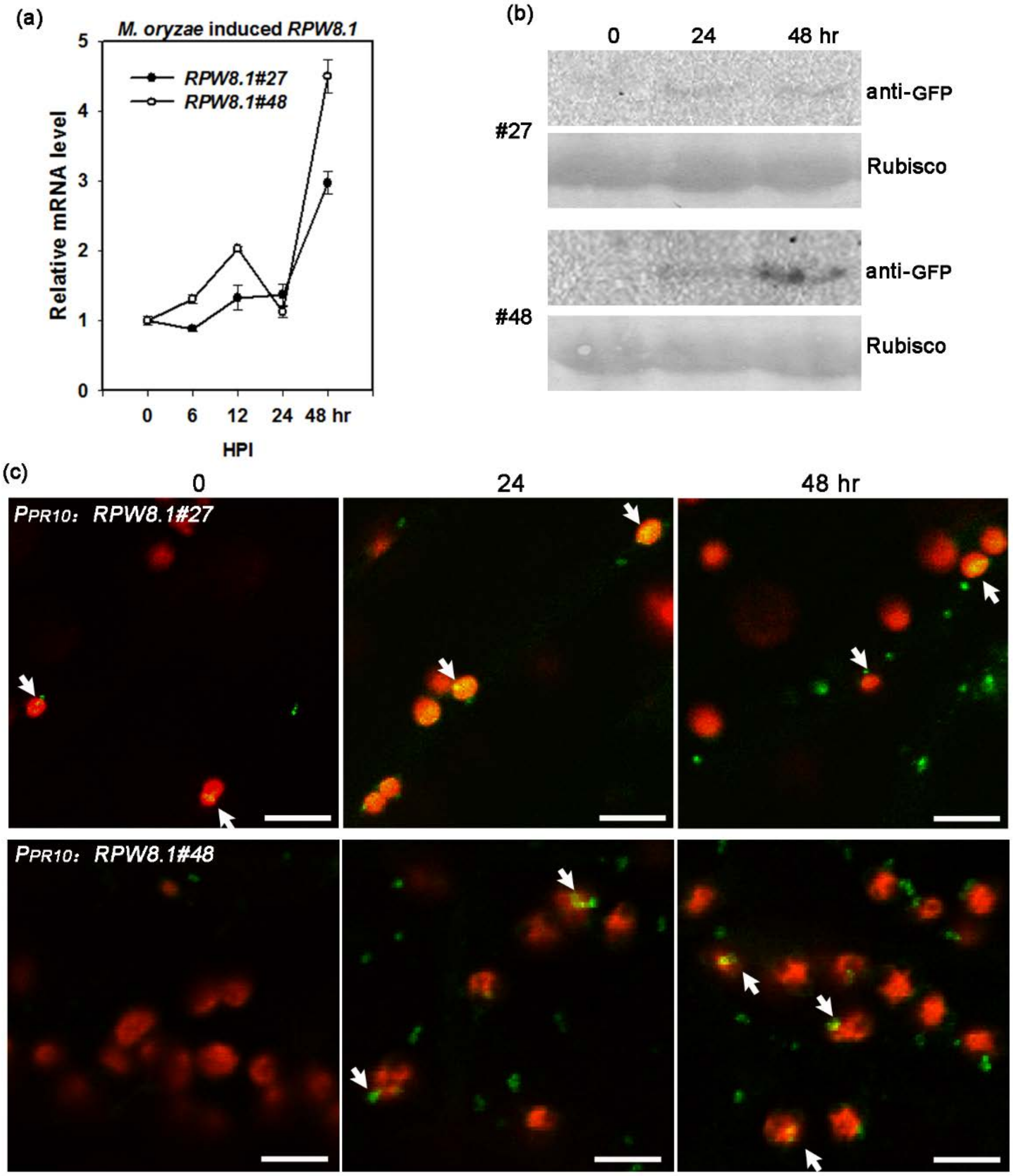

Figure S6 *P. oryzae* up-regulates the expression of *RPW8.1* in transgenic rice plants. (a) Expression pattern of *RPW8.1* in the indicated transgenic rice lines upon *P. oryzae* infection. The leaves of four-week-old seedlings were spray-inoculated with the *P. oryzae* strain Guy11, and total RNA was extracted at the indicated time points for quantitative RT-PCR analysis. Relative mRNA level was normalized to that of non-inoculated seedlings. Error bars indicate standard deviation (n=3). The experiments were repeated two times with similar results. (b) Western blot analysis shows the protein accumulation of RPW8.1-YFP in two transgenic rice lines upon *P. oryzae* infection. Four-week-old seedlings were spray-inoculated with the *P. oryzae* strain Guy11. Protein was extracted at the indicated time points for western blot analysis with anti-GFP serum. Rubisco was stained by Ponceau S as loading control. (c) Subcellular accumulation of RPW8.1-YFP in the indicated transgenic rice lines upon infection of *P. oryzae*. The leaf sheaths from seven-week-old plants were inoculated with the *P. oryzae* strain Guy11, and the subcellular accumulation pattern of RPW8.1-YFP in leaf sheath cells was acquired by laser scanning confocal microscopy at the indicated time points. RPW8.1-YFP was pseudo-colored green, and the auto-fluorescence from chloroplast/plastid was pseudo-colored red. Note that RPW8.1-YFP was often associated with chloroplasts/plastids (arrows). Size bar, 10 μm.

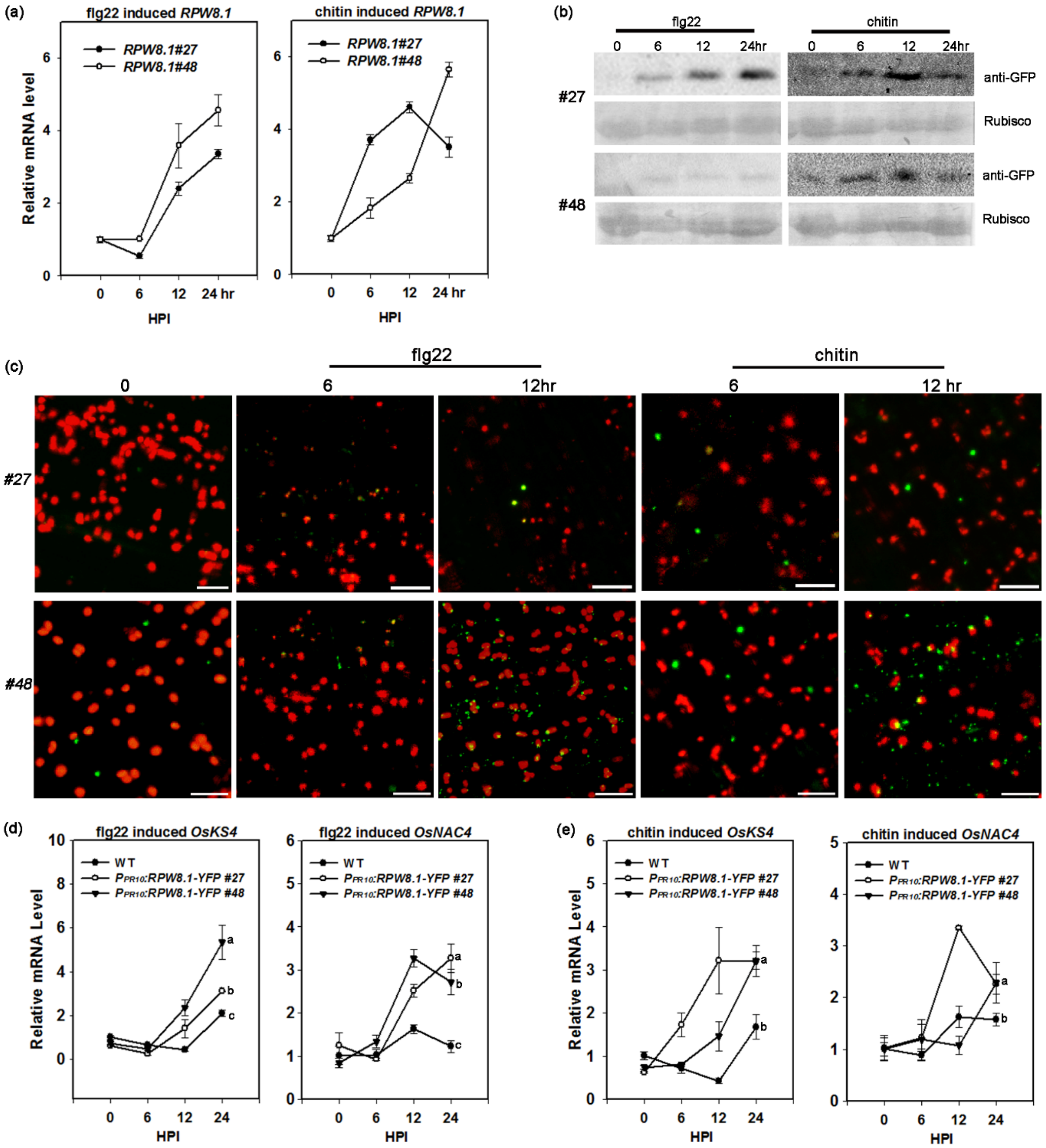

(a)
flg22 induced RPW8.1
RPW8.1#27
RPW8.1#48
Relative mRNA level
HPI
0 6 12 24 hr
chitin induced RPW8.1
RPW8.1#27
RPW8.1#48
(b)
flg22
chitin
0 6 12 24hr
#27
#48
anti-GFP
Rubisco
(c)
flg22
chitin
0
6
12hr
12 hr
#27
#48
(d)
flg22 induced OsKS4
flg22 induced OsNAC4
WT
P_{PR10}:RPW8.1-YFP #27
P_{PR10}:RPW8.1-YFP #48
Relative mRNA Level
HPI
(e)
chitin induced OsKS4
chitin induced OsNAC4

Figure S7 PAMPs up-regulate expression of *RPW8.1* in transgenic rice plants. (a) Quantitative RT-PCR data show the expression pattern of *RPW8.1* in rice transgenic lines upon application of flg22 or chitin. Leaves from five-week-old plants were incubated in water containing flg22 or chitin. Total RNA was extracted at the indicated time points for quantitative RT-PCR analysis. Relative mRNA level was normalized to that in untreated plants. Error bars indicate standard deviation (SD, n=3). The experiments were repeated two times with similar results. (b) Western blot analysis shows the accumulation of RPW8.1-YFP in the indicated transgenic lines upon application of flg22 or chitin. Five-week-old plants were incubated in water containing flg22 or chitin. Protein was extracted at the indicated time points for western blot analysis with anti-GFP serum. Rubisco was stained with Ponceau S as loading control. (c) Representative confocal images show the subcellular accumulation of RPW8.1-YFP in the indicated transgenic rice lines. Leaf sheaths from seven-week-old plants were inoculated in water containing flg22 or chitin. The subcellular accumulation pattern of RPW8.1-YFP in leaf sheath cells was acquired by laser scanning confocal microscopy at the indicated time points. RPW8.1-YFP was pseudo-colored green, and the auto-fluorescence from chloroplast was pseudo-colored red. Size bar, 10 μm. (d, e) Expression pattern of PAMPs-induced basal defense-related genes *OsNAC4* and Os*KS4* in the indicated transgenic rice lines upon application of flg22 (d) or chitin (e). Total RNA obtained from (a) was used to explore the transcription of the two indicated genes. Relative mRNA levels were normalized to that in untreated wild type (WT) TP309 plants. Error bars indicate SD (n=3). Different letters at 24 HPI indicate significant differences ($P < 0.01$) as determined by a one-way ANOVA followed by post hoc Tukey HSD analysis. Similar results were obtained in two independent experiments. HPI, hours post incubation.
